# Genome Mining of Terpene Synthases from Fourteen *Streptomyces* Strains

**DOI:** 10.3390/microorganisms13071479

**Published:** 2025-06-25

**Authors:** Yuanyuan Li, Xi Xiang, Zhiyuan Ren, Rui Wang, Minghui Xie, Gen Li, Xiaohui Yan, Zhilong Zhao, Zixin Deng, Min Xu, Anwei Hou

**Affiliations:** 1 Tianjin Institute of Industrial Biotechnology, Chinese Academy of Sciences, Tianjin 300308, Chinawangruizzy@tib.cas.cn (R.W.); zxdeng@sjtu.edu.cn (Z.D.); 2 Haihe Laboratory of Synthetic Biology, Tianjin 300308, China; 3 School of Chinese Materia Medica, Tianjin University of Traditional Chinese Medicine, Tianjin 301617, China; 4 College of Medical, Linyi University, Linyi 276000, China

**Keywords:** terpene synthases, *Streptomyces*, enzyme catalysis, terpenoids

## Abstract

Terpenoids are the most structurally diverse class of natural products (NPs). Despite their abundance, the functional diversity of bacterial terpene synthases (TPSs), particularly from *Streptomyces* species, remains largely unexplored. In this study, fourteen *Streptomyces* strains were subjected to genome sequencing and bioinformatic analysis to systematically mine class I TPSs. A total of forty-eight TPSs were identified and categorized through phylogenetic analysis, and five representative TPSs distantly related to known TPSs were selected for functional investigation. Biochemical assays revealed that TAC28_6116 is a sesquiterpene synthase producing thujopsan-2β-ol (**1**) and thujopsene (**2**), marking the first report of thujopsan-2β-ol production from a bacterial source. TAC49_7078 is a diterpene synthase responsible for the formation of *ent*-phomacta-1(15),3,7-triene (**3**). Notably, TAC43_2999 was identified as a novel sesterterpene synthase that produced compound **5** in vitro, while the generation of a previously undescribed compound **6**, sestermalaysiene, was exclusively detected during in vivo fermentation using the engineered *Escherichia coli* chassis optimized for terpenoid biosynthesis. Structural elucidation of sestermalaysiene was supported by nuclear magnetic resonance (NMR) analysis and quantum chemical calculations. Its formation might proceed via a rare [4 + 2] cycloaddition mechanism. Overall, this work expands our knowledge of the catalytic diversity of bacterial TPSs and offers promising biocatalysts for terpenoid engineering and discovery.

## 1. Introduction

Terpenoids are recognized as the largest and most structurally diverse class of natural products (NPs), with over 100,000 compounds identified to date [1,2]. They play essential roles in natural ecosystems, functioning as hormones, pigments, and defense compounds. For example, gibberellins are diterpenoid phytohormones that regulate plant growth [3], while carotenoids such as lycopene contribute to the pigmentation of fruits [4]. In addition, terpenoids have significant applications in pharmaceuticals (e.g., the anticancer agent paclitaxel) [5,6], fragrances (e.g., menthol) [7,8], and biofuels (e.g., farnesene) [9,10], thus contributing to a wide range of applications in industrial and medical fields. Traditionally, terpenoids have been predominantly studied from plants, animals, and fungi [2,5,11]. Key enzymes, such as terpene synthases (TPSs), catalyze the cyclization of linear precursors into structurally diverse skeletons, forming the basis for the remarkable chemical diversity observed in terpenoids. TPSs are fundamentally categorized into two classes based on catalytic mechanism [12,13]. Class I TPSs, characterized by a conserved DDXXD motif and NSE/DTE triad, coordinate a magnesium ion cluster that facilitates the departure of the pyrophosphate group from the substrate. This pyrophosphate elimination generates an allylic carbocation, initiating cyclization cascades. Class I TPSs can be classified based on the chain length of their characteristic substrates: monoterpene synthases (geranyl pyrophophate, GPP, C_10_), sesquiterpene synthases (farnesyl prophosphate, FPP, C_15_), diterpene synthases (geranylgeranyl pyrophoshpate, GGPP, C_20_), sesterterpene synthases (geranylfarnesyl pyrophosphate, GFPP, C_25_) and even triterpene synthases (hexaprenyl pyrophosphate, HexPP, C_30_) [14]. In contrast, class II TPSs employ a diagnostic DXDD motif to protonate epoxide rings or alkenyl double bonds of the substrates, directly forming carbocation intermediates that drive terpene skeleton cyclization. Functionally, class II TPSs are defined by their catalytic role and products, rather than their linear precursors.

More recently, bacteria, particularly *Streptomyces* species, have been recognized as an important source of terpenoid biosynthesis, with numerous TPS genes identified in their genomes [1,15,16]. According to data from the InterPro database (https://www.ebi.ac.uk/interpro/; accessed on 9 April 2025), nearly half of the annotated bacterial class I TPSs originate from *Streptomyces*. However, despite the abundance of TPS genes present in the bacterial genomes, the majority of TPSs remain functionally uncharacterized. This gap is particularly notable for bacterial diterpene and sesterterpene synthases [1,17], where documented examples remain limited. Such scarcity constrains our understanding of both the structural diversity and biosynthetic potential of bacterial terpenoids. Therefore, functional characterization of these underexplored TPSs is essential for unlocking the biosynthetic capacities of *Streptomyces* and advancing microbial NP research.

The advent of high-throughput sequencing technologies has revolutionized the field of genomics, making genome sequencing more accessible and cost-effective [18]. These technological advancements provide unprecedented opportunities to systematically explore microbial genomes and identify novel biosynthetic genes. To leverage these advancements, we conducted a comprehensive analysis of TPSs across the draft genomes of fourteen *Streptomyces* strains. Specifically, we aimed to systematically identify novel class I TPSs from these *Streptomyces* genomes and functionally characterize their enzymatic activities. By integrating genome mining with experimental validation, we seek to expand the current knowledge of bacterial terpenoid biosynthesis and uncover TPSs with potential applications in biotechnology and NP discovery. Our findings are expected to improve the understanding of terpenoid biosynthetic diversity and provide new insights into the metabolic potential of *Streptomyces*, thereby contributing to the broader exploration and utilization of microbial NPs.

## 2. Materials and Methods

### 2.1. General Experimental Procedures

Gas chromatography–mass spectrometry (GC-MS) analysis was carried out on an Agilent 8860-5977C GC-MS system (Agilent, Santa Clara, CA, USA) using an HP-5MS capillary column (30 m × 0.25 mm, 0.25 μm film thickness). The GC conditions were as follows: inlet pressure, 22.153 psi; helium flow rate, 33.93 mL min^−1^; injection volume, 1 μL; and a temperature program of 50 °C (held for 5 min), ramped at 10 °C/min to 300 °C, followed by an isothermal hold at 300 °C for 5 min. The helium was used as the carrier gas at a flow rate of 1.2 mL min^−1^. The mass spectrometry (MS) settings were as follows: ion source temperature, 230 °C; transfer line temperature, 250 °C; quadrupole temperature, 150 °C.

Nuclear magnetic resonance (NMR) spectra were recorded on a Quantum-I 400 MHz spectrometer (Q.One Instruments, Wuhan, China) and a Bruker AVANCE III 600 MHz spectrometer (Bruker, Billerica, MA, USA) using standard 5 mm NMR tubes. Samples were dissolved in deuterated benzene (C_6_D_6_), and chemical shifts (*δ*) were referenced to the residual solvent peaks (*δ*_H_ 7.16 ppm, *δ*_C_ 128.06 ppm). All 1D and 2D NMR experiments were acquired using standard pulse sequences under conventional acquisition conditions.

Optical rotation values were measured using an MCP 150 automatic polarimeter (Anton Paar, Graz, Austria). Ultrapure water was produced using a UPR-11-10TNZP ultrapure water system (UPR^®^ series; Sichuan Youpu Ultrapure Technology Co., Ltd., Chengdu, China). All chemical reagents and fermentation media were purchased from commercial sources and used without further purification.

### 2.2. Isolation and Identification of Streptomyces Strains

Soil samples were collected from Changbai Mountain, China, and 10 g of soil powder were suspended in 90 mL of sterile distilled water in a sterile Erlenmeyer flask. The suspension was subjected to ultrasonic treatment at 50 Hz for 30 min to facilitate homogenization. The treated suspension was serially diluted (10^−2^ to 10^−5^), and 200 μL aliquots of each dilution were spread onto Gauze’s Synthetic Medium No. 1 plates (composition per liter: 20 g soluble starch, 1 g KNO_3_, 0.5 g K_2_HPO_4_, 0.5 g MgSO_4_·7H_2_O, 0.1 g FeSO_4_·7H_2_O, 100 mg nystatin, 20 mg nalidixic acid, and 15 g agar; pH 7.2). The plates were incubated at 30 °C for 7 days. Single colonies with characteristic actinomycete morphology were selected and purified by streaking onto ISP2 agar medium (composition per liter: 4 g yeast extract, 10 g malt extract, 4 g glucose, and 15 g agar; pH 7.2) and incubated at 30 °C for 3–5 days (File S1, Figure S1). For DNA extraction, a single colony was inoculated into 2 mL of TSBY medium (composition per liter: 30 g tryptic soy broth and 5 g yeast extract) and cultured at 30 °C with shaking at 250 rpm until the culture became turbid. Mycelia were harvested by centrifugation at 13,300 rpm for 5 min, washed once with sterile distilled water, and centrifuged again. The pellet was resuspended in 100 μL of PrepMan^TM^ Ultra reagent (Cat. No. 4318930, Thermo Fisher Scientific, Waltham, MA, USA), vortexed thoroughly, and heated at 95 °C for 15 min, followed by centrifugation at 13,300 rpm for 10 min to collect the supernatant containing genomic DNA. The 16S rRNA gene was amplified using the universal primers 27F (5′-AGAGTTTGATCCTGGCTCAG-3′) and 1492R (5′-GGTTACCTTGTTACGACTT-3′) in a 50 μL PCR reaction mixture comprising 2 μL genomic DNA (20–50 ng/μL), 1 μL of each primer (10 μM), 25 μL Taq DNA polymerase premix (Accurate Biology Co., Ltd. [AG Bio], Changsha, China), and sterile water. PCR amplification was performed under the following conditions: 94 °C for 4 min; 35 cycles of 98 °C for 10 s, 55 °C for 30 s, and 72 °C for 1 min; followed by a final extension at 72 °C for 10 min. Amplified products were verified by 1% agarose gel electrophoresis and sequenced bidirectionally by Sanger sequencing (GENEWIZ, Suzhou, China). The obtained sequences were assembled and manually corrected to generate high-quality consensus sequences, which were submitted to the NCBI GenBank database for BLAST analysis (https://blast.ncbi.nlm.nih.gov/Blast.cgi; accessed on 28 March 2025) against type strains. Phylogenetic identification was carried out based on the closest matches. The 16S rRNA gene was further confirmed by draft genome sequences described below. For long-term storage, the strains were preserved by mixing cell suspensions with 40% sterile glycerol at a 1:1 ratio and stored at −80 °C.

### 2.3. Genomic DNA Extraction, Sequencing, and Annotation

A 2 mL aliquot of fresh *Streptomyces* seed culture was inoculated into 50 mL of TSBY medium supplemented with 0.4–0.5% glycine and incubated at 28–30 °C with shaking at 250 rpm for 48 h. Cells were harvested by centrifugation (7000 rpm, 10 min) and 0.5 g of the resulting wet cell pellet was transferred into a 2 mL microcentrifuge tube. The cells were washed twice with 1 mL H_2_O and resuspended in 5 mL of SET buffer (5 mM NaCl, 25 mM EDTA, 20 mM Tris-HCl, pH 7.5). Lysozyme (CM0082, AG Bio) was added to a final concentration of 1 mg/mL (250 µL), and the mixture was incubated at 37 °C for 10 min. Following cell wall digestion, 5 M NaCl (2 mL) and chloroform (7 mL) were added. The mixture was gently mixed until an opaque emulsion formed and was centrifuged at 7000 rpm for 15 min at 4 °C. The aqueous (upper) phase was collected, and chloroform extraction was repeated once. An equal volume of pre-chilled isopropanol was added to the aqueous phase to precipitate genomic DNA. The resulting DNA pellet was washed twice with 70% ethanol (5 mL) and air-dried in a biosafety cabinet. The DNA was resuspended in 500 µL of TE buffer (10 mM Tris-HCl, 1 mM EDTA, pH 8.0). RNase A (5 µL; AG12002, AG Bio) was added to a final concentration of 1% and incubated at 37 °C for 1 h to remove RNA. Subsequently, Proteinase K (12.5 µL; AG12004, AG Bio) was added to a final concentration of 0.5 mg/mL and the solution was incubated at 55 °C for 1 h. The sample was then extracted with an equal volume (500 µL) of chloroform, followed by a second round of precipitation using 3 M sodium acetate (100 µL, pH 5.2) and isopropanol (600 µL). After washing twice with 70% ethanol (1 mL) and drying, the DNA was dissolved in 500 µL of TE buffer.

Whole-genome sequencing was performed using the Illumina HiSeq 2500 platform (Genoscreen, Lille, France) with a coverage depth of approximately 100×. Raw reads were quality-filtered using the Trimmomatic software package (version 0.33), and de novo assembly of the filtered reads was conducted using SPAdes v3.6.2 (File S1, Table S1). Gene prediction was carried out using Prodigal v2.6.3, and the predicted genes were translated into protein sequences based on the codon usage of *Streptomyces*. All genome sequencing and annotation procedures were performed by Biomarker Technologies, Inc. (Beijing, China).

### 2.4. Polygenetic Tree Analysis

Multiple sequence alignments were performed using the Clustal Omega (v1.2.2) algorithm implemented in Geneious Prime (version 2025.1.2, Biomatters Ltd., Auckland, New Zealand). Sequences were grouped in order of similarity for alignment, with the number of refinement iterations set to 30. Both the initial and refinement guide trees were generated based on the evaluation of the full distance matrix. Subsequently, a phylogenetic tree was constructed from the alignment results using the Neighbor-Joining method with the Jukes–Cantor genetic distance model. No outgroup was specified, and bootstrap analysis with 1000 replicates was conducted to assess statistical support for the tree topology. The resulting topologies were automatically sorted, and a consensus tree was generated based on a topology threshold of 35%. All other parameters were set to their default values unless otherwise specified. The generated tree was further refined and visualized using the Interactive Tree of Life (iTOL) online tool (https://itol.embl.de/; accessed on 28 March 2025).

### 2.5. Gene Cloning and Expression

TPS genes were amplified from genomic DNA using gene-specific forward and reverse primers containing homologous arms (Table 1), and were subsequently assembled into the linearized pET-28a(+) plasmid (digested with *HindIII* and *NdeI*) via Gibson assembly. Plasmids were extracted from 5 mL liquid cultures of the corresponding *Escherichia coli* DH5α strains using the SanPrep Column Plasmid MiniPreps Kit (Vazyme, Nanjing, China). PCR amplifications were performed on an Eppendorf thermal cycler (Thermo Fisher Scientific, Waltham, MA, USA) using KOD One™ PCR Master Mix (Toyobo, Osaka, Japan). DNA concentrations were measured using a NanoDrop 2000c Microvolume UV-Vis spectrophotometer (Thermo Fisher Scientific). Sanger sequencing, as well as the synthesis of primers and other oligonucleotides, was performed by Tsingke Biotechnology Co., Ltd. (Beijing, China).

For in vivo expression of TPSs, the corresponding TPS genes were amplified using the primer pairs F2/R2 (Table 1). Depending on the predicted function of each TPS, a corresponding prenyltransferase gene—either FPP synthase [19] (for sesquiterpenes), GGPP synthase [20] (for diterpenes), or GFPP synthase [20] (for sesterterpenes)—was co-amplified using gene-specific primers. These gene fragments were then assembled into the linearized pET-21a plasmid (digested with *HindIII* and *NdeI*) via Gibson assembly. The resulting recombinant plasmids were transformed into *E. coli* competent cells harboring an engineered complete mevalonate (MVA) pathway [19]. The transformed strains were cultured at 37 °C until the optical density at 600 nm (OD_600_) reached 0.6, followed by induction with 0.4 mM isopropyl β-d-1-thiogalactopyranoside (IPTG). After induction, the cultures were incubated at 18 °C for 20 h and subsequently fermented at 30 °C for an additional 3 days. Approximately 6 L of culture was used for metabolite extraction. The fermentation broth was extracted with acetone, and the crude extract was purified using silica gel chromatography followed by semi-preparative high-performance liquid chromatography (HPLC).

### 2.6. Protein Purification and Biochemical Assays

Cell pellets collected from 250 mL cultures were resuspended in 15 mL of binding buffer (50 mM Tris-HCl, 300 mM NaCl, 20 mM imidazole, 1 mM MgCl_2_, pH 7.4, 4 °C) and lysed by ultrasonication. The lysate was centrifuged at 8000 rpm for 60 min to remove cell debris. The resulting supernatant was applied to a Ni^2+^-NTA affinity chromatography column (GenScript, Nanjing, China), followed by washing with binding buffer (2 × 2 mL) and elution of the target protein using 2 mL of elution buffer (50 mM Tris-HCl, 300 mM NaCl, 250 mM imidazole, 1 mM MgCl_2_, pH 7.4, 4 °C). The purity and concentration of the eluted protein fractions were analyzed by SDS-PAGE (File S1, Figure S2) and Bradford assay (C503031, Sangon Bioceth Co., Ltd., Shanghai, China).

For enzyme assays, purified proteins (0.3 mL each) were used at the following concentrations: TAC43-2999 (ca. 5.6 mg/mL), TAC49-7078 (ca. 3.0 mg/mL), TAC28-6116 (ca. 2.8 mg/mL), and TAC43-2367 (ca. 0.9 mg/mL). Each reaction mixture contained 0.3 mL of enzyme solution, 0.2 mL of substrate solution (1.0 mg/mL GFPP [15], GGPP [21], FPP, or GPP in 25 mM NH_4_HCO_3_), and 0.5 mL of incubation buffer (50 mM Tris-HCl, 4 mM MgCl_2_, 10% glycerol, pH 7.4). Reactions were incubated overnight at 30 °C. Enzymatic products were extracted with 0.3 mL of hexane, and then analyzed by GC-MS. For enzyme activity assays, reactions were carried out in a total volume of 1 mL containing 50 mM Tris-HCl buffer (pH adjusted to 7.4), 4 mM MgCl_2_, 10% (*v*/*v*) glycerol, 5 μM purified enzyme, and 0.4 mg of substrate. The reaction mixtures were incubated at 30 °C for 3 h, extracted with 0.3 mL of hexane, and analyzed by GC-MS.

### 2.7. Quantum Chemistry Calculations

Density functional theory (DFT) calculations were conducted using Gaussian 16 [22]. To assess the correctness of the elucidated structure, an initial conformational search was carried out using Spartan’24 (Wavefunction Inc., Irvine, CA, USA) with the corrected MMFF force field. Conformers with Boltzmann populations above 0.01 were retained for further analysis. Geometry optimizations and frequency calculations were performed at the B3LYP/6-31G(d) level to confirm local minima. Subsequently, Gibbs free energy was used to compute Boltzmann distributions, and conformers with populations exceeding 2% were selected for ^13^C NMR shielding tensor calculations using the GIAO method at the mPW1PW91/6-31G(d,p) level, with the polarizable continuum model (PCM) applied. Chemical shifts were referenced to tetramethylsilane (TMS), and the final ^13^C NMR values were obtained as Boltzmann-weighted averages. A linear correlation analysis between the calculated and experimental NMR data was conducted to evaluate the accuracy of the elucidated structure.

## 3. Results

### 3.1. Genome Mining for TPSs in the Draft Genomes of Fourteen Streptomyces Strains

**Figure 1:**
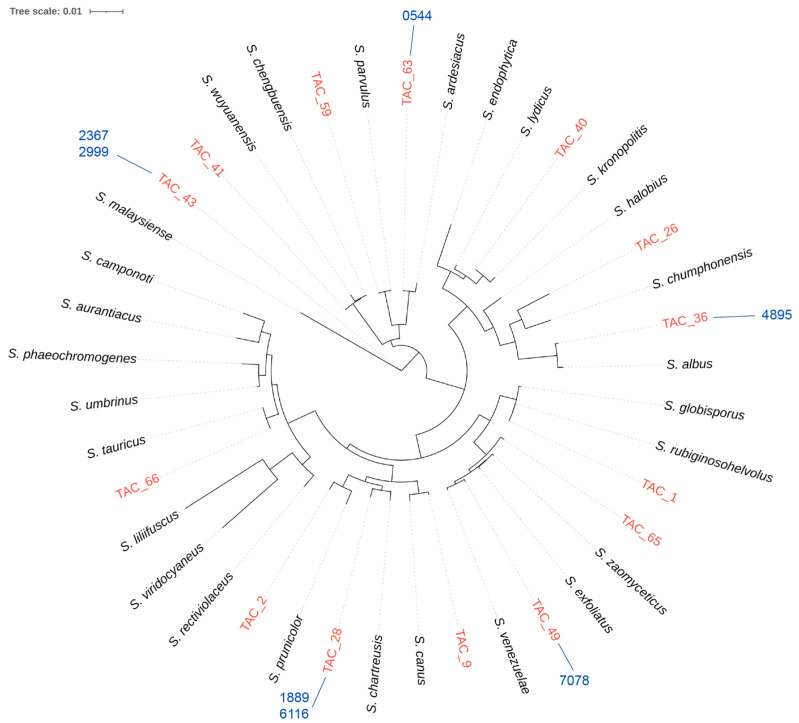
Phylogenetic analysis based on 16S rRNA sequences of 14 isolated *Streptomyces* strains and their closely related reference strains. The corresponding sequences are provided in File S2, and the reference strain information is listed in Table S2 (File S1). TPSs selected for further study are annotated in blue alongside the corresponding strains.

Strains exhibiting the characteristic colony morphology of Actinomycetes were isolated from soil samples and confirmed as belonging to the genus of *Streptomyces* through 16S rRNA sequencing. A total of 14 distinct *Streptomyces* species were identified (Figure 1) and subjected to genome sequencing using the Illumina HiSeq platform. The sequencing cost for each sample was approximately USD 56, cheaper than synthesizing a 1 kb gene (~USD 70). To ensure high sequencing accuracy, a 100× sequencing depth was employed, resulting in over 99% coverage of the respective strains’ genomes. This extensive coverage provides a strong foundation for further exploration and mining of TPSs.

Gene annotation was subsequently performed, and the predicted genes were translated into protein sequences based on the codon usage of *Streptomyces*. TPSs were identified using HMMER [23] with the HMM profile PF19086, which corresponds to class I TPSs in the InterPro database. All 48 mined class I TPSs are summarized in Supplementary File (File S3).

### 3.2. Polygenetic Analysis of TPSs

**Figure 2:**
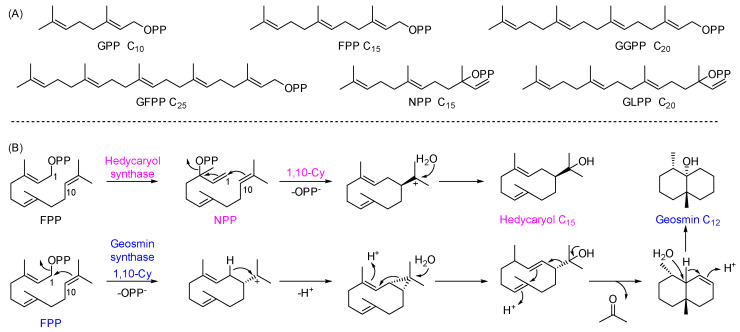
Substrates and description conventions of TPSs. (**A**) Chemical structures of typical TPS substrates, including GPP (C_10_), FPP (C_15_), GGPP (C_20_), and GFPP (C_25_). The FPP isomer NPP and GGPP isomer GLPP are also shown. (**B**) Description convention for TPSs. TPSs are described as “Ca/b/Xc-d/Product name,” where “Ca” denotes the number of carbon atoms in the product, “b” the number of rings, and “Xc-d” the initial cyclization mode (e.g., “N1-10” indicates 1,10-cyclization of NPP). If the detailed mechanism is unclear, it is omitted. The enzyme name or main product name is then used. In this figure, “C15/1/N1-10/Hedycaryol synthase” represents a TPS that catalyzes the 1,10-cyclization of NPP to form the monocyclic sesquiterpene alcohol hedycaryol, while “C12/2/F1-10/Geosmin synthase” represents a TPS catalyzing the formation of the bicyclic compound geosmin.

**Figure 3:**
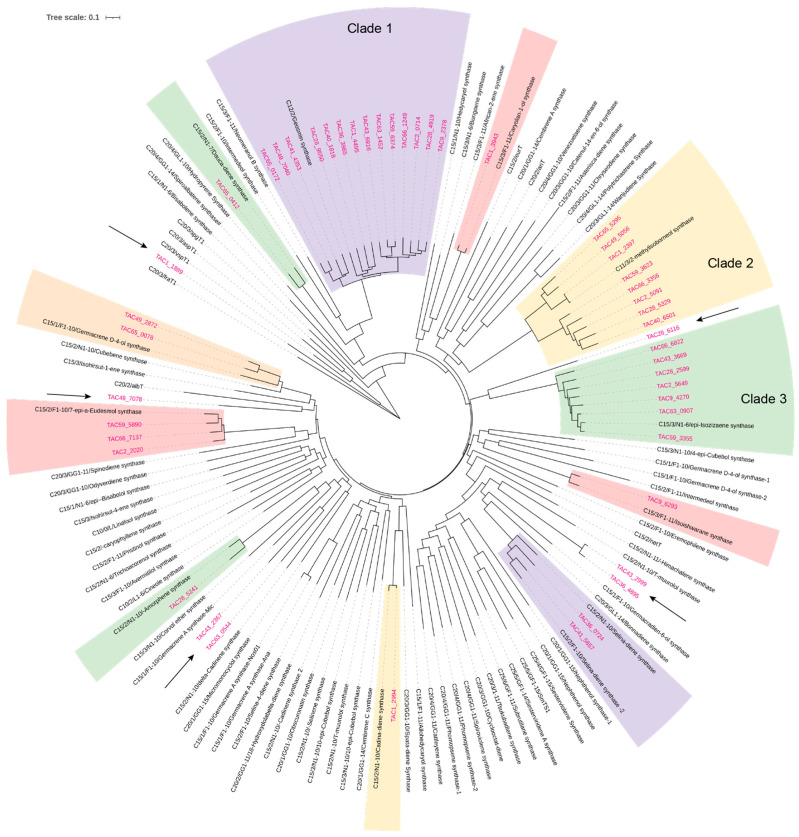
Phylogenetic analysis of 48 TPSs identified from the 14 strains, together with functionally characterized reference TPSs. The corresponding sequences of the 48 TPSs are provided in the Supplementary File S3. TPSs showing low sequence identity to characterized reference TPSs are indicated by arrows.

We collected approximately 90 sequences of reported typical class I TPSs and briefly annotated their major product information in the sequence names [17,21,24]. For instance, “C15/1/N1-10/Hedycaryol synthase” [25] indicates that the enzyme primarily produces a sesquiterpene (C_15_) with a monocyclic skeleton formed via a 1,10-cyclization of nerolidyl pyrophosphate (NPP) (Figure 2). NPP and geranyllinalyl pyrophosphate (GLPP) are isomers of FPP and GGPP, respectively [17,26]. We then performed a phylogenetic analysis of the 48 mined TPSs together with the collected reference enzymes and inferred their possible functions based on their sequence identities (Figure 3).

In the phylogenetic tree, Clade 1 corresponds to “C12/2/F1-10/Geosmin synthase”. Geosmin synthase is a bifunctional terpene synthase that utilizes FPP as its substrate but uniquely removes a C_3_ unit during the cyclization process, representing a distinctive catalytic mechanism among characterized TPSs (Figure 2) [27]. Geosmin is known for imparting the typical earthy odor characteristic of *Streptomyces*. All 14 strains possess one Clade 1-associated protein sequence, indicating a conserved enzymatic function among these strains. In addition, eight strains possess protein sequences with high sequence identity to “C11/3/2-methylisoborneol synthase,” which utilizes 2-methylated GPP as its substrate (Figure 3, Clade 2) [28]. Similarly, seven protein sequences from distinct strains belong to Clade 3, represented by the known enzyme “C15/3/N1-6/epi-Isozizaene synthase” [29]. The lowest sequence identity within this clade is approximately 72%, suggesting that these enzymes likely share the same function. Besides these three clades, several other clades contain proteins with very high sequence identity to characterized TPSs. For example, TAC_6293 shares 87% identity with “C15/3/F1-11/Isoishwarane synthase” [30], and TAC_5241 shows 79% identity with “C15/2/N1-10/Amorphene synthase” [31]. Based on these similarities, the functions of 41 mined TPSs can be tentatively annotated. We then focused on characterization of the remaining proteins, whose sequences show low identity to characterized ones, aiming to uncover potentially novel TPSs.

### 3.3. Characterization of TPSs

Two of the unannotated TPSs, TAC43_2999 and TAC36_4895, share a high sequence identity (93%). Therefore, TAC43_2999 was selected for further analysis. In the case of TAC28_1889, it is positioned within a clade containing enzymes such as “C20/3/vspT1”. These enzymes are typically found in genomic regions adjacent to class II TPSs, which catalyze the cyclization of GGPP into intermediates such as *ent*-copalyl diphosphate (*ent*-CPP). To further explore the potential function of TAC28_1889, we analyzed its surrounding genomic context and identified a nearby gene encoding a protein with high similarity to a known *ent*-CPP synthase [32]. Moreover, sequence alignment revealed that TAC28_1889 shares 85% identity with pimara-9(11),15-diene synthase [33], indicating its involvement in the same diterpene biosynthetic pathway. As a result, this enzyme was not selected for further investigation. The genes encoding four other enzymes (TAC28_6116, TAC63_0544, TAC49_7078, and TAC43_2367), as well as TAC43_2999 were cloned into the pET-28a(+) vector with an N-terminal His-tag fused to the enzyme, enabling purification via Ni^2+^-NTA affinity chromatography. For convenience, the enzymes are hereafter referred to by the last four digits of their locus tags (e.g., TAC43_2999 is referred to as 2999).

**Figure 4:**
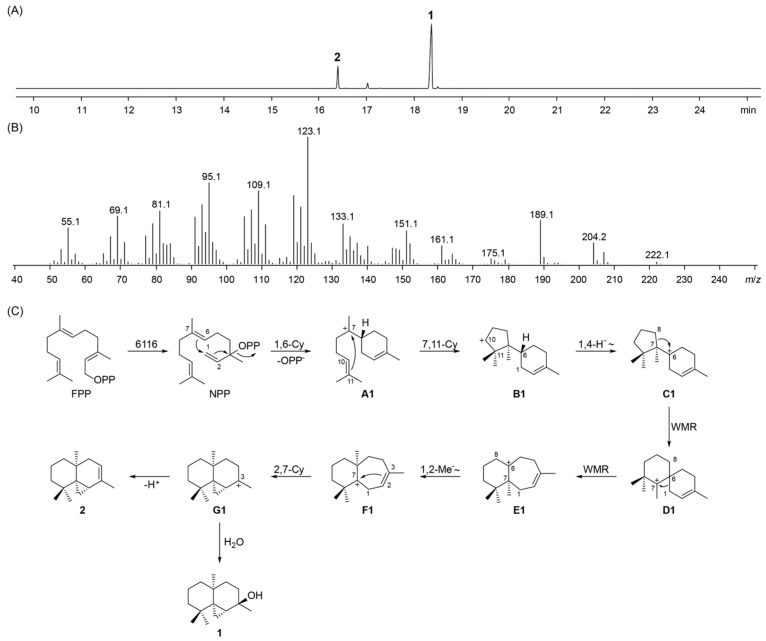
GC-MS analysis of the product generated by 6116 and its proposed biosynthetic mechanism. (**A**) Total ion chromatogram (TIC) of products from 6116 incubated with FPP. (**B**) Electron ionization (EI) mass spectrum of **1**. (**C**) Proposed biosynthetic pathway of compounds **1** and **2** catalyzed by 6116.

After enzyme purification and in vitro assays with four native substrates (GPP, FPP, GGPP, and GFPP) of TPSs, 6116 was identified as a sesquiterpene synthase, producing one terpene alcohol as the major product (Figure 4A,B). To obtain a sufficient amount of the products for NMR analysis, the gene of 6116 were cloned into a pET-21a(+) plasmid along with a gene encoding FPP synthase, and the constructed plasmid was transformed into an engineered *E. coli* chassis with an exogenous mevalonate pathway [19]. Following fermentation, purification, and compound characterization, thujopsan-2β-ol (**1**) [34] (File S1, Figures S3 and S4) was identified as the major product and thujopsene (**2**) [35,36] (File S1, Figures S5 and S6) was detected as a minor one. Both compounds have been reported as components of essential oils from various plants, such as *Origanum calcaratum* [34] and *Cananga odorata* [37]. To the best of our knowledge, 6116 represents the first bacterial-origin enzyme capable of producing these two compounds. In addition, its product formation pathway could be proposed by analogy to the biosynthetic mechanisms characterized in fungi [38]. In this pathway, FPP first undergoes isomerization to generate NPP, which subsequently undergoes a 1,6-cyclization to form intermediate **A1** (Figure 4C). Then, **A1** experiences a 7,11-cyclization to yield **B1**, followed by a 1,4-hydride shift to form **C1**. Successive Wagner–Meerwein rearrangements (WMRs) convert **C1** into **D1** and subsequently into **E1**. Following this, a 1,2-methyl shift generates intermediate **F1**, which then undergoes a 2,7-cyclization to produce **G1**. The fate of **G1** determines the final product outcome: nucleophilic attack by water affords compound **1**, while deprotonation results in the formation of compound **2**. Moreover, NMR analysis revealed that compound **1** could spontaneously convert into compound **2** in CDCl_3_. This transformation is likely facilitated by the slightly acidic nature of CDCl_3_, which promotes the elimination of the hydroxyl group from compound **1**.

**Figure 5:**
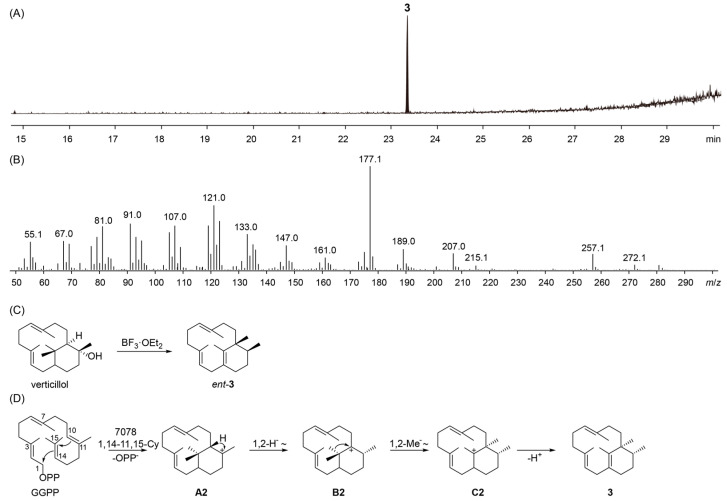
GC-MS analysis of the product generated by 7078 and its proposed biosynthetic mechanism, in comparison with the chemical synthesis route of *ent*-**3**. (**A**) Extracted ion chromatogram (EIC) at *m*/*z* 177, corresponding to the base peak of **3** in the EI mass spectrum. (**B**) EI mass spectrum of **3**. (**C**) Chemical synthesis of *ent*-**3** from verticillol using BF_3_·OEt_2_ as a catalyst. (**D**) Proposed biosynthetic pathway of compound **3** catalyzed by 7078.

The expression of 0544 in *E. coli* BL21(DE3) failed to yield detectable protein, as evidenced by the absence of a distinct band on SDS-PAGE after purification. Additionally, no enzymatic activity was detected in the eluted fractions subjected to functional assays. As the GC content differs significantly between *Streptomyces* and *E. coli*, codon optimization of the 0544 gene could be a potential strategy for improving protein expression and will be considered in future work. In contrast, although the expression level of 7078 was relatively low, a clear protein band was observed after purification. In vitro enzymatic assays further revealed that this enzyme functions as a diterpene synthase (Figure 5A,B). Subsequent in vivo heterologous expression of 7078 in the engineered *E. coli* platform led to the identification of **3** (File S1, Figures S7 and S8) as the major product. Compound **3** exhibits identical NMR data to phomacta-1(15),3,7-triene (*ent*-**3**), which was previously chemically synthesized from verticillol using BF_3_·OEt_2_ as a catalyst (Figure 5C) [39]. *Ent*-**3** is also known as a side product of CsCTS, a diterpene synthase from *Cephalotaxus sinensis* [40]. To date, no bacterial enzyme has been reported to produce compound **3**. Moreover, its specific rotation ([α]_D_^20^ = −9, *c* 0.1, CH_2_Cl_2_; File S1) is opposite to that of the reported *ent*-**3** ([α]_D_^20^ = +49, *c* 0.33, CH_2_Cl_2_) [40], indicating that compound **3** is its enantiomer. Its structure was elucidated as shown in Figure 5D. Such enantiomeric variations are commonly observed for terpenoids derived from different biological sources [17]. A biosynthetic mechanism analogous to that of CsCTS has been proposed for compound **3**. The proposed pathway is initiated by a 1,14-10,15 cyclization to generate intermediate **A2**, which subsequently undergoes a 1,2-hydride shift to afford carbocation intermediate **B2**. This is followed by a 1,2-methyl shift to yield intermediate **C2**, and final deprotonation of **C2** results in the formation of compound **3** (Figure 5D).

**Figure 6:**
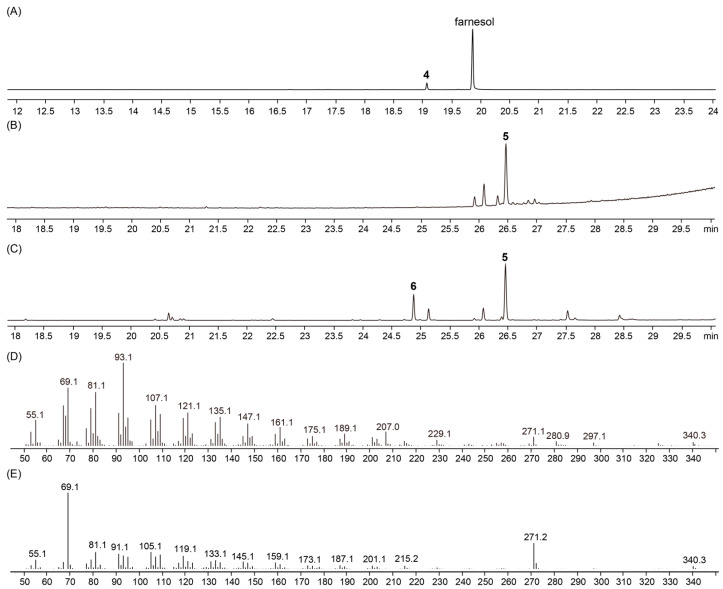
GC-MS analysis of the products generated by 2367 and 2999. (**A**) TIC of the products from 2367 incubated with FPP. (**B**) TIC of the products from 2999 incubated with GFPP. (**C**) TIC of the fermentation products from *E. coli* harboring 2999. (**D**) EI mass spectrum of **5.** (**E**) EI mass spectrum of **6**.

The expression level of 2367 in *E. coli* BL21(DE3) was very low, and only a faint protein band was observed on SDS-PAGE after purification. However, in vitro assays confirmed that the protein functions as a T-muurolol synthase, whose catalytic activity and product profile have been previously investigated by us in detail [41]. The enzymatic product **4** was unambiguously identified using Agilent MassHunter Qualitative Analysis software (version 10.0) by comparison with both NIST database entries and the mass spectrum of an authentic standard compound. The major peak in the TIC was identified as farnesol (Figure 6A), possibly formed by a contaminating phosphatase or other enzyme in the eluted protein solution that hydrolyzed FPP. Therefore, we did not conduct further experiments to isolate or characterize this compound. For 2999, the expression was successful (File S1, Figure S2), and in vitro assays revealed that it is a sesterterpene synthase (Figure 6B). Further heterologous expression in the *E. coli* strain led to the isolation of **5** and **6** as the two major products (Figure 6C). However, **6** was not detected in the in vitro reactions, suggesting that it might have been produced during a fermentation or extraction process. 

**Figure 7:**
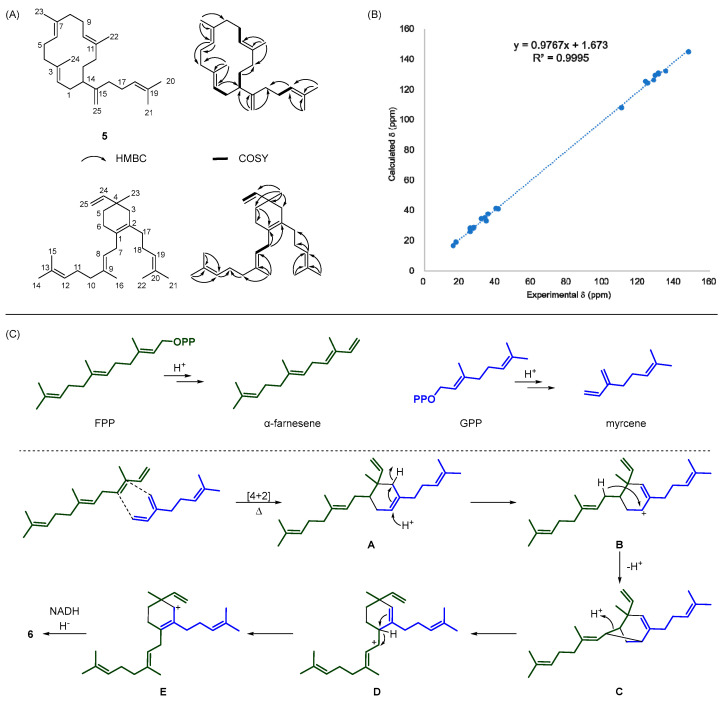
Structural elucidation of compounds **5** and **6**, and validation of compound **6** by DFT-calculated ^13^C NMR data, along with its proposed biosynthetic mechanism. (**A**) Key HMBC and COSY correlations for the structure elucidation of compounds **5** and **6**. (**B**) Linear correlation between the DFT-calculated and experimental ^13^C NMR chemical shifts of compound **6**. (**C**) Proposed biosynthetic mechanism of compound **6** via a [4 + 2] cycloaddition.

Through comprehensive analysis of one- and two-dimensional NMR spectra, along with their MS spectra (Figure 6D,E), the structures of **5** (File S1, Figures S9–S15) and **6** (File S1, Figures S16–S22) were elucidated (Figure 7A, Table 2). The structure of **5** has previously only been reported in the context of chemical synthesis [42], and has not been isolated from natural sources. Given its monocyclic structure, it is likely derived from the 1,14-cyclization of GFPP followed by deprotonation to yield the final product. In contrast, compound **6** represents a novel structure whose formation cannot be rationalized by conventional cyclization pathways from GFPP. Due to its unusual structure and the significant overlap of multiple signals—which made certain signals difficult to distinguish in the HSQC and HMBC spectra (e.g., *δ*_C_ 131.22 and 131.17, *δ*_C_ 17.77 and 17.76)—we performed density functional theory (DFT)-based NMR calculations to verify the correctness of the structure (File S1, Table S3, Files S4 and S5). As shown in Figure 7B, the calculated NMR data exhibited an excellent linear correlation with the experimental results (*R*^2^ = 0.9995), strongly supporting the structural assignment of **6**. In consideration of the close phylogenetic relationship between strain TAC43 and *S. malaysiensis*, compound **6** was therefore designated as sestermalaysiene. Given its unique structure, its formation is proposed to involve a [4 + 2] cycloaddition between myrcene and farnesene [43], both of which might be generated during the fermentation process under high precursor supply (Figure 7C). During the *E. coli* fermentation, acetic acid may accumulate, promoting the pyrophosphate elimination from FPP and GPP to yield farnesene and myrcene, respectively. These two precursors could then undergo a [4 + 2] cycloaddition under the fermentation conditions to form intermediate **A**. Subsequently, under acidic conditions, intermediate **A** may be protonated to generate cation **B**, which could rearrange via intermediate **C** to form cation **D**. Cation **D** might then undergo a hydride shift coupled with double bond migration, leading to intermediate **E**. This intermediate could accept a hydride ion from NADH, yielding the final product **6**. The formation of this product appears to require a specific microenvironment to facilitate the complex reaction sequence. Nevertheless, the low specific optical rotation of compound **6**, [α]20D = −1.6 (c 0.13, CH_2_Cl_2_), suggests that the product is likely a racemic mixture, implying that the entire process could be non-enzymatic.

To further assess the catalytic performance of the characterized TPSs, specific activities were determined and expressed as units per μmol of protein (U·μmol^−1^ protein), with one unit (U) defined as the enzymatic conversion of 1 μmol of substrate into product per hour. TAC28_6116 exhibited a significantly higher specific activity of 71.42 U·μmol^−1^ protein, whereas TAC43_2367, TAC49_7078, and TAC43_2999 displayed much lower activities of 0.54, 0.53, and 0.91 U, respectively. These findings underscore the considerable variability in the catalytic efficiencies among the characterized TPSs. The notably high specific activity of 6116 suggests its potential for scalable biosynthesis of sesquiterpene products, whereas the relatively low activities of 2367, 7078, and 2999 indicate that further optimization—such as codon optimization, protein engineering, or improvements in expression systems—may be necessary to enhance their catalytic performance for broader biotechnological applications.

## 4. Conclusions and Discussion

In summary, this study systematically explored the diversity and catalytic potential of class I TPSs from fourteen *Streptomyces* strians through draft genome sequencing, phylogenetic analysis, and functional characterization. A total of 48 TPSs were identified, among which most could be tentatively annotated based on sequence similarity to known enzymes. Notably, all these strains harbored geosmin synthase, while eight of them additionally possessed 2-methylisoborneol (2-MIB) synthase. These widespread geosmin and 2-MIB synthases, which are responsible for the characteristic earthy odor of soil, are believed to play important ecological roles in mediating microbial–arthropod interactions and promoting the dispersal of *Streptomyces* spores in natural environments [32].

More importantly, five TPSs representing potentially novel catalytic functions were selected for detailed biochemical investigation. Among them, TAC28_6116 was identified as a bacterial sesquiterpene synthase capable of producing thujopsan-2β-ol and thujopsene, compounds commonly found in plant essential oils but not reported from bacterial sources. This enzyme may serve as a valuable component for producing plant essential oil compounds in engineered microbial chassis, providing a sustainable platform for the biosynthesis of these valuable natural products. TAC49_7078 was functionally characterized as a diterpene synthase responsible for generating *ent*-phomacta-1(15),3,7-triene (**3**), whose biosynthetic pathway resembles that of CsCTS. In addition, TAC43_2999 was identified as a novel sesterterpene synthase that catalyzes the formation of compound **5** in vitro, while in vivo it yields both compounds **5** and **6**. Notably, compound **6** exhibits an unprecedented structure, presumably arising from a biosynthetic [4 + 2] cycloaddition. The formation of compound **6** appears to be particularly unusual, suggesting that the underlying biosynthetic mechanism may require further investigation. Despite these advances, several limitations of this study should be noted. TAC63_0544 showed no detectable expression, and TAC43_2367 exhibited low expression levels. Additionally, the possibility of non-enzymatic transformations of compound **6** cannot be ruled out. Future work will aim to improve the expression of these TPSs by codon optimization and the use of fusion tags, as well as to elucidate the detailed biosynthetic mechanisms of compound **6**, thereby enhancing the robustness of our findings.

Overall, these findings expand our understanding of the catalytic diversity of bacterial TPSs and provide novel biocatalysts for future efforts in terpenoid pathway engineering and NP discovery. In particular, exploration of adjacent tailoring genes, such as cytochrome P450s, may facilitate the biosynthesis of an even more diverse array of terpenoid structures. Furthermore, the bioactivities of these compounds warrant further investigation to evaluate their potential pharmaceutical or industrial applications.

## Figures and Tables

**Table 1:** Primers used in this study.

Primer Name	Sequence (5′ to 3′)
2999-F	CTGGTGCCGCGCGGCAGCCATATGATGAGCAGCACATCAGGT
2999-R	GGTGCTCGAGTGCGGCCGCAAGCTTTCAGCAGTGGTCCCAC
7078-F	GCCGCGCGGCAGCCATATGATGCCGATCGATGTGGACT
7078-R	TCGAGTGCGGCCGCAAGCTTTCAACGGGCTTCTCCGATCT
0554-F	GCCGCGCGGCAGCCATATGATGCGAGAAGGGGCG
0554-R	TCGAGTGCGGCCGCAAGCTTGCGGCTGCCGCAC
2367-F	CTGGTGCCGCGCGGCAGCCAATGGAGTTGATGCTGCCG
2367-R	TGGTGCTCGAGTGCGGCCGCATGATCGAGGCAGGTCTCA
6116-F	GCCGCGCGGCAGCCATATGATGAGGGACGACCGCTACTACCAC
6116-R	TCGAGTGCGGCCGCAAGCTTTCAAGCTTGTTCCAGCCGG
2999-F2	CTTTAAGAAGGAGATATACATATGAGCAGCACATCAGG
2999-R2	GCATTAGTATCCCCCTTAGATGCTTAGTTTGAGCTCGAATTCGGATCCTCAGCAGTGGTCCCACC
7078-F2	TAAAACTAAGCATCTAAGGGGGATACTAATGCCGATCGATGTGGACT
7078-R2	GTGGTGGTGCTCGAGTGCGGCCGCATCAACGGGCTTCTCCGAT
6116-F2	GCATCTAAGGGGGATACTAATGAGGGACGACCGCTA
6116-R2	GTTAGCAGCCGGATCTCAAGCTTGTTCCAGCC
GFPPS-2999-F	GCTGAGGATCCGAATTCGAGCTCAAACTAAGCATCTAAGGGGGATACTAATGCCCGTAAAAGTCCACG
GFPPS-2999-R	CTCGAGTGCGGCCGCAAGCTTTACAGGTTCTCTCCAGCATATCC
GGPPS-7078-F	CGCTACTGCTCACCTCATTTAAGGAGGTTTTTTATGTCTACTGAAACG
GGPPS-7078-R	AACAGAAAAATCTGGATTTGATACAAAGTCTACCTCAACACCAAC
FPPS-6116-F	CTAGAAATAATTTTGTTTAACTTTAAGAAGGAGATATACATATGATGGATTTTCCCCAACAGCT
FPPS-6116-R	CCCTTAGATGCTTAGTTTTACTCTAAGCTTATTTATTACGTTGGATGATGTAGT

**Table 2:** NMR data of compounds **5** and **6** in C_6_D_6_ (600 MHz).

No. ^1^	5		6	
	*δ*_C_ ^2^	*δ*_H_ ^2^	*δ*_C_ ^2^	*δ*_H_ ^2^
1	33.63	2.25 (m, 1H); 2.07 (m, 1H)	129.28	-
2	124.79	5.33 (m, 1H)	128.68	-
3	134.82	-	41.64	2.06 (m, 1H); 1.84 (m, 1H)
4	39.40	2.17 (m, 2H)	35.67	-
5	25.35	2.27 (m, 1H); 2.15 (m, 1H)	34.60	1.49 (m, 1H); 1.40 (m, 1H)
6	126.51	5.09 (td, *J* = 5.8, 2.7 Hz, 1H)	27.49	2.18 (m, 1H); 2.05 (m, 1H)
7	133.43	-	32.05	2.85 (t, *J* = 6.2 Hz, 2H)
8	39.90	2.11 (m, 2H)	123.98	5.26 (m, 1H)
9	24.20	2.17 (m, 2H)	135.17	-
10	122.30	5.27 (m, 1H)	40.27	2.10 (m, 2H)
11	133.88	-	27.15	2.18 (m, 2H)
12	34.58	2.07 (m, 1H); 2.00 (m, 1H)	124.91	5.22 (m, 1H)
13	29.25	1.87 (m, 1H); 1.53 (m, 1H)	131.22	-
14	45.39	2.24 (m, 1H)	25.90	1.67 (s, 3H)
15	153.52	-	17.76	1.55 (s, 3H)
16	34.82	2.12 (m, 2H)	16.21	1.65 (s, 3H)
17	27.26	2.26 (m, 2H)	33.96	2.17 (m, 2H)
18	125.03	5.26 (m, 1H)	27.68	2.18 (m, 2H)
19	131.31	-	125.22	5.28 (m, 1H)
20	25.85	1.68 (s, 3H)	131.17	-
21	17.76	1.70 (s, 3H)	25.91	1.69 (s, 3H)
22	108.85	4.94 (m, 2H)	17.77	1.60 (s, 3H)
23	18.22	1.61 (s, 3H)	25.80	1.03 (s, 3H)
24	15.37	1.55 (s, 3H)	148.02	5.87 (dd, *J* = 17.5, 10.8 Hz, 1H)
25	15.61	1.56 (s, 3H)	110.55	5.03 (dd, *J* = 17.5, 1.5 Hz, 1H); 4.99 (dd, *J* = 10.8, 1.5 Hz, 1H)

^1^ Atom numbering corresponds to Figure 6. ^2^ Chemical shifts δ are reported in ppm. Multiplicities are given as follows: s = singlet, d = doublet, t = triplet, m = multiplet.

## Data Availability

The original contributions presented in the study are included in the article/Supplementary Material, further inquiries can be directed to the corresponding authors.

## Supplementary Materials

The following supporting information can be downloaded at: https://www.mdpi.com/article/10.3390/microorganisms13071479/s1. File S1: Supporting information including contigs, draft genome sizes, NMR spectra of isolated compounds, and characterization data of known compounds. File S2: 16S rRNA gene sequences of the 14 isolated strains. File S3: Sequences of 48 TPSs identified from the 14 isolated strains. File S4: Generated conformers for ^13^C NMR calculations. File S5: The calculated ^13^C NMR data for the filtered conformers. Figure S1: Morphology of fourteen isolated strains on ISP2 agar plates; Table S1: Contig length, number of contigs, and number of TPSs in the 14 isolated strains; Table S2: Information on selected strains used for phylogenetic analysis based on the 16S rRNA gene; Figure S2: SDS-PAGE analysis of the purified protein; Figure S3: ^1^H NMR spectrum of **1** (600 MHz, C_6_D_6_); Figure S4: ^13^C NMR spectrum of **1** (150 MHz, C_6_D_6_); Figure S5: ^1^H NMR spectrum of **2** (600 MHz, CDCl_3_); Figure S6: ^13^C NMR spectrum of **2** (150 MHz, CDCl_3_); Figure S7: ^1^H NMR spectrum of **3** (600 MHz, C_6_D_6_); Figure S8: ^13^C NMR spectrum of **3** (150 MHz, C_6_D_6_); Figure S9: ^1^H NMR spectrum of **5** (600 MHz, C_6_D_6_); Figure S10: ^13^C NMR spectrum of **5** (150 MHz, C_6_D_6_); Figure S11: ^13^C DEPT135 spectrum of **5** (150 MHz, C_6_D_6_); Figure S12: ^1^H-^1^H COSY spectrum of **5** (C_6_D_6_); Figure S13: HSQC spectrum of **5** (C_6_D_6_); Figure S14: HMBC spectrum of **5** (C_6_D_6_); Figure S15: NOESY spectrum of **5** (C_6_D_6_); Figure S16: ^1^H NMR spectrum of **6** (600 MHz, C_6_D_6_); Figure S17: ^13^C NMR spectrum of **6** (150 MHz, C_6_D_6_); Figure S18: ^13^C DEPT135 spectrum of **6** (150 MHz, C_6_D_6_); Figure S19: ^1^H-^1^H COSY spectrum of **6** (C_6_D_6_); Figure S20: HSQC spectrum of **6** (C_6_D_6_); Figure S21: HMBC spectrum of **6** (C_6_D_6_); Figure S22: NOESY spectrum of **6** (C_6_D_6_); Table S3: Calculated energies and Boltzmann populations of **6** conformers in benzene.
