# Supplementary material for: Genome Mining of Terpene Synthases from Fourteen Streptomyces Strains
Source: Microorganisms. 2025 Jun 25;13(7):1479. doi: 10.3390/microorganisms13071479 (PMC12298776; doi:10.3390/microorganisms13071479)
Supplement: Supplementary file 1 [file microorganisms-13-01479-s001.zip › microorganisms-3655725-supplementary.pdf]

Supporting information

Table of Contents

1.	Figure S1	S2
2	Table S1	S3
3.	Table S2	S4
4.	Figure S2	S5
5.	Characterization data for the isolated compounds	S6
6.	Copies of NMR spectra	S7–S26
7.	Table S3	S27

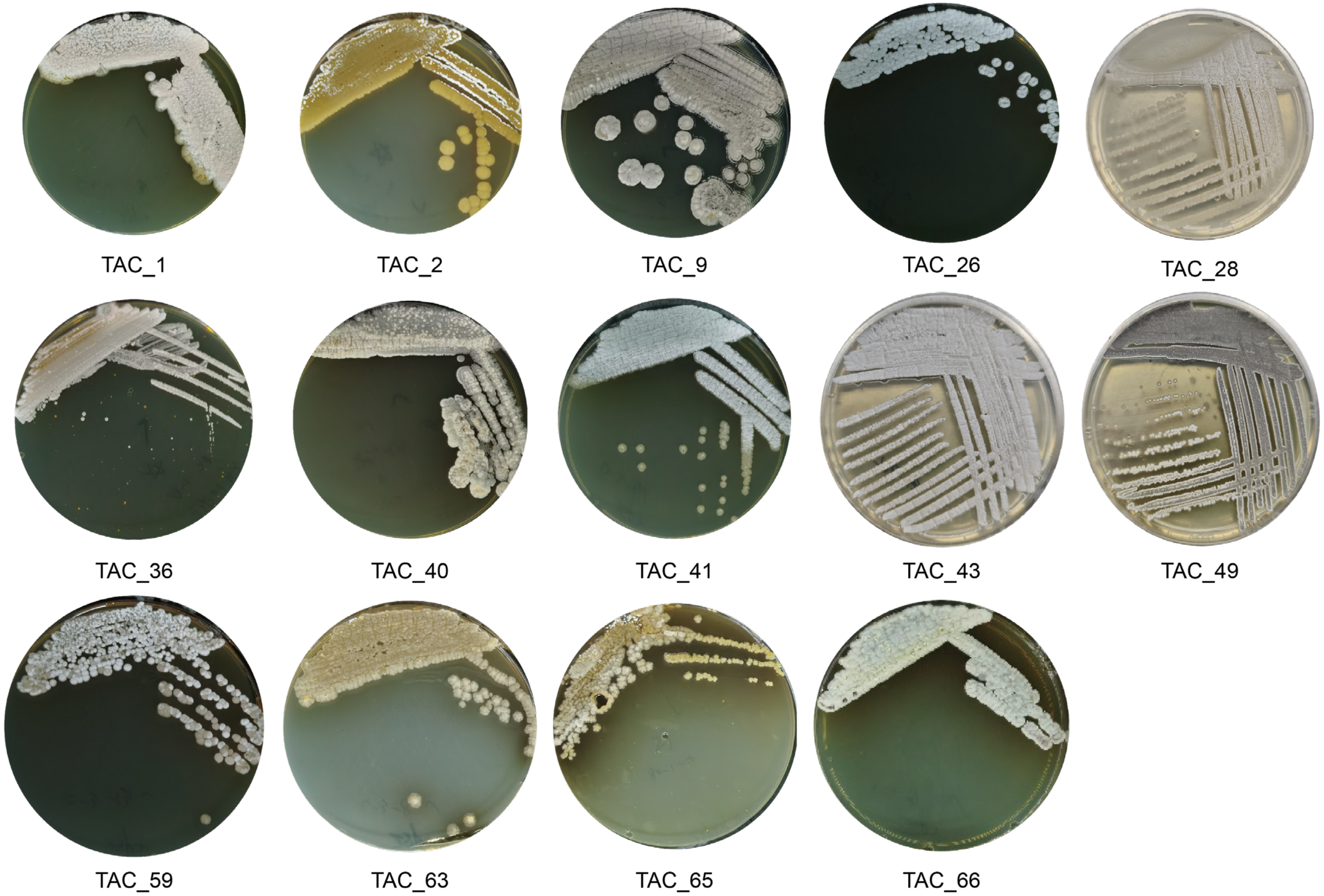

Figure S1. Morphology of fourteen isolated strains on ISP2 agar plates.

Table S1 Contig length, number of contigs, and number of TPSs in the 14 isolated strains.

NO.	Strain NO.	Contig Length (bp) [1]	Contig number	TPS number [2]
1	TAC1	8,222,773	51	5
2	TAC2	10,315,260	58	4
3	TAC9	10,172,247	37	3
4	TAC26	17,089,994	1,196	1
5	TAC28	10,456,627	74	5
6	TAC36	7,809,079	52	3
7	TAC40	7,762,502	119	2
8	TAC41	7,928,076	65	2
9	TAC43	7,891,981	44	4
10	TAC49	8,257,888	60	4
11	TAC59	7,692,847	52	4
12	TAC63	7,999,470	70	3
13	TAC65	7,568,820	51	4
14	TAC66	10,304,746	74	4

[1] Contig length refers to the total length of all contigs in the assembly.
[2] Number of Class I TPSs.

Table S2. Information on selected strains used for phylogenetic analysis based on the 16S rRNA gene.

Entry No.	*Streptomyces* species	Strain No.
1	*S. albus*	**DSM 40313**
2	*S. ardesiacus*	**NRRL B-1773**
3	*S. aurantiacus*	**NBRC 15418**
4	*S. camponoti*	**2C-SSA16-1**
5	*S. canus*	**NBRC 12872**
6	*S. chartreusis*	**NBRC 12753**
7	*S. chengbuensis*	**HUAS CB01**
8	*S. chumphonensis*	**KK1-2**
9	*S. endophytica*	**HNM0140**
10	*S. exfoliatus*	**NBRC 13191**
11	*S. globisporus*	**NRRL B-2872**
12	*S. halobius*	**3_2**
13	*S. kronopolitis*	**NEAU-ML8**
14	*S. liliifuscus*	**ZYC-3**
15	*S. lydicus*	**ATCC 25470**
16	*S. malaysiense*	**MUSC 136**
17	*S. parvulus*	**NBRC 13193**
18	*S. phaeochromogenes*	**NBRC 3180**
19	*S. prunicolor*	**NBRC 13075**
20	*S. rectiviolaceus*	**NRRL B-16374**
21	*S. rubiginosohelvolus*	**NBRC 12912**
22	*S. tauricus*	**JCM 4837**
13	*S. umbrinus*	**NBRC 13091**
24	*S. venezuelae*	**JCM 4526**
25	*S. viridocyaneus*	**T20**
26	*S. wuyuanensis*	**FX61**
27	*S. zaomyceticus*	**NRRL B-2038**

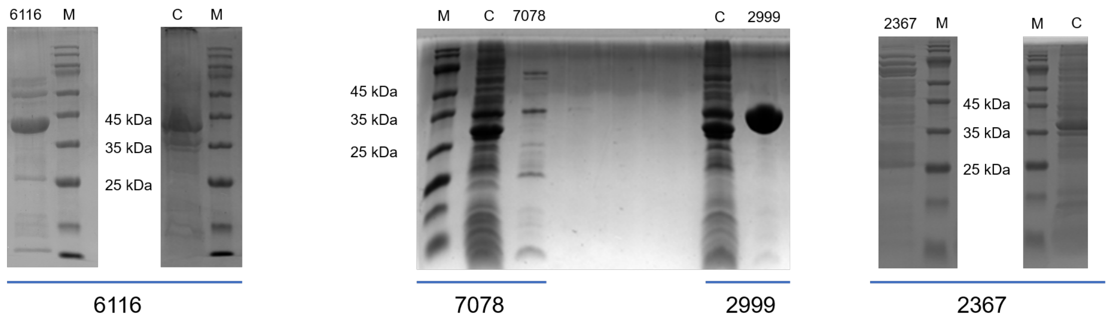

Figure S2. SDS-PAGE analysis of the purified protein. All proteins were fused with an N-terminal His-tag. 6116: 44.2 kD; 7078: 44.2 kD; 2999: 43.0 kD; 2367: 35.4 kD. M indicates the protein marker; C indicates the resuspended pellet of cell debris obtained by centrifugation after ultrasonication.

Characterization data for the isolated compounds

Compound **1**, colorless oil. $[\alpha]_D^{20}$ = –48.5 (*c* 0.13, CH_2Cl_2) ^1H NMR (600 MHz, C_6D_6): δ = 1.79 – 1.66 (m, 1H), 1.47 (td, *J* = 13.0, 4.1 Hz, 1H), 1.43 – 1.30 (m, 5H), 1.33 (s, 3H), 1.17 (dd, *J* = 10.1, 5.8 Hz, 1H), 1.15 – 1.06 (m, 2H), 1.06 (d, *J* = 0.9 Hz, 3H), 0.99 (s, 3H), 0.75 – 0.66 (m, 1H), 0.47 (s, 3H), 0.40 (t, *J* = 5.4 Hz, 1H), 0.17 (dd, *J* = 10.2, 5.1 Hz, 1H) ppm. ^{13}C NMR (151 MHz, C_6D_6) δ 66.90, 41.08, 36.60, 34.84, 34.54, 34.11, 33.69, 33.67, 32.14, 32.02, 29.58, 29.45, 27.31, 19.21, 9.01.

Compound **2**, colorless oil. $[\alpha]_D^{20}$ = –146.0 (*c* 0.1, CH_2Cl_2) ^1H NMR (600 MHz, $CDCl_3$): δ = 5.03 (dq, *J* = 6.5, 1.5 Hz, 1H), 1.83 – 1.81 (m, 3H), 1.81 – 1.72 (m, 2H), 1.50 – 1.36 (m, 4H), 1.28 – 1.19 (m, 2H), 1.12 (s, 3H), 1.11 (s, 3H), 1.09 (q, *J* = 3.5 Hz, 1H), 0.74 – 0.66 (m, 2H), 0.61 (s, 3H) ppm. ^{13}C NMR (151 MHz, $CDCl_3$): δ = 135.70, 114.58, 41.33, 40.52, 36.73, 35.10, 34.01, 31.59, 29.19, 28.73, 26.93, 23.62, 22.39, 19.74, 11.43 ppm.

Compound **3**, colorless oil. $[\alpha]_D^{20}$ = –9 (*c* 0.1, CH_2Cl_2) ^1H NMR (600 MHz, C_6D_6) δ 5.09 (ddd, *J* = 11.8, 3.8, 1.7 Hz, 1H), 4.85 (ddt, *J* = 10.6, 2.7, 1.4 Hz, 1H), 3.15 (t, *J* = 12.3 Hz, 1H), 2.33 (dddd, *J* = 14.9, 12.1, 10.6, 4.1 Hz, 1H), 2.30 – 2.15 (m, 2H), 2.10 – 1.94 (m, 4H), 1.89 – 1.73 (m, 3H), 1.69 – 1.61 (m, 1H), 1.61 (t, *J* = 1.3 Hz, 3H), 1.54 (q, *J* = 1.3 Hz, 3H), 1.53 – 1.44 (m, 1H), 1.43 – 1.36 (m, 1H), 1.35 (t, *J* = 2.0 Hz, 3H), 1.33 – 1.25 (m, 1H), 0.84 (d, *J* = 7.0 Hz, 3H), 0.83 (s, 3H).. ^{13}C NMR (151 MHz, C_6D_6) δ = 133.70, 132.99, 131.94, 130.69, 127.51, 124.92, 41.33, 38.99, 34.93, 34.73, 33.60, 32.77, 31.28, 28.01, 27.04, 22.05, 16.62, 16.24, 15.79, 15.36 ppm.

Compound **5**, colorless oil. $[\alpha]_D^{20}$ = –94 (*c* 0.1, CH_2Cl_2). For NMR data, see Ttable 2 in main text.

Compound **6**, colorless oil. $[\alpha]_D^{20}$ = –1.6 (*c* 0.13, CH_2Cl_2). For NMR data, see Ttable 2 in main text. HRMS (EI): calcd. for $C_{25}H_{40}$ $[M]^+$ 340.3130, found 340.3137.

Copies of NMR spectra

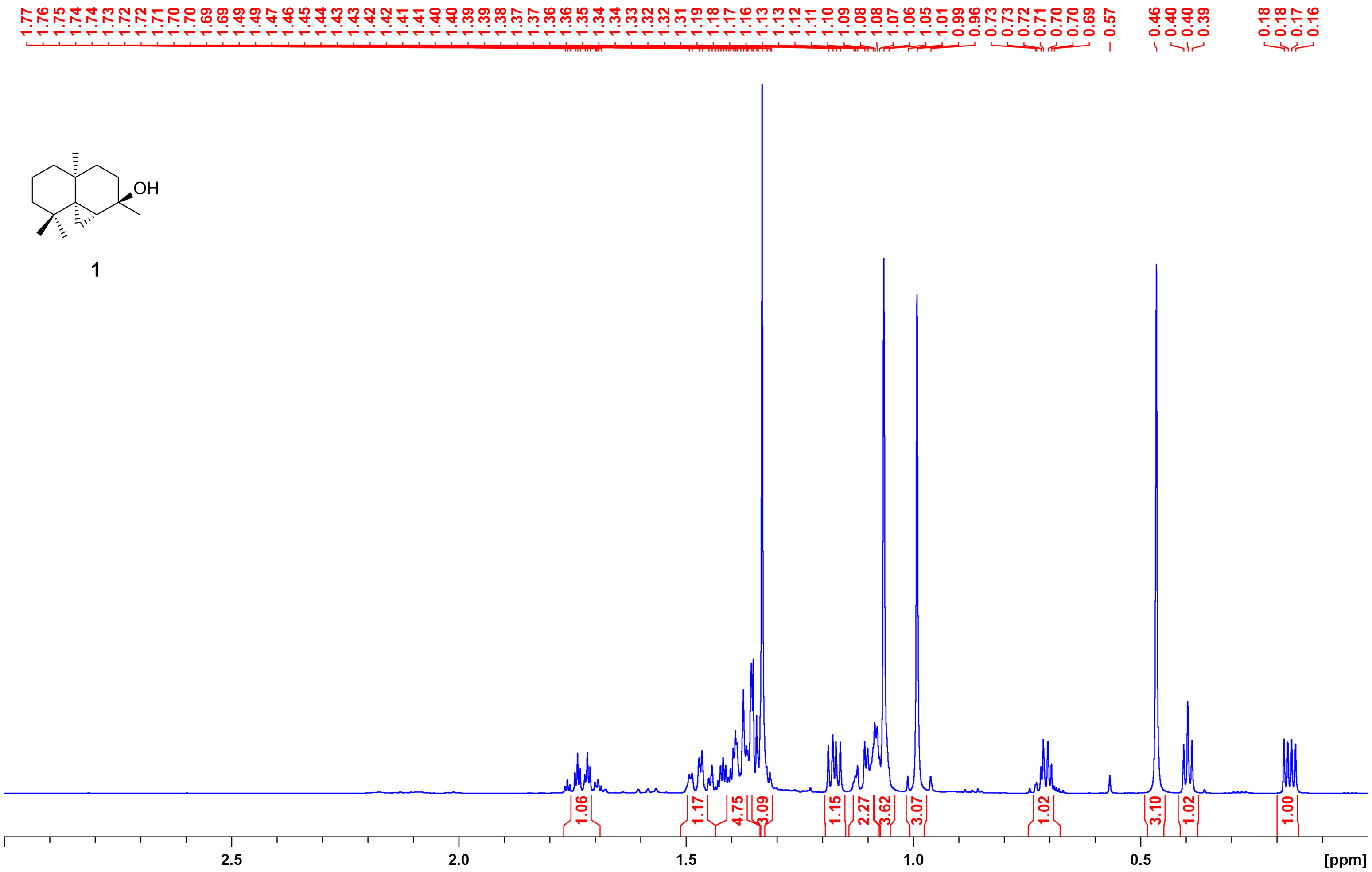

Figure S3. ^{1}H NMR spectrum of **1** (600 MHz, C_6D_6).

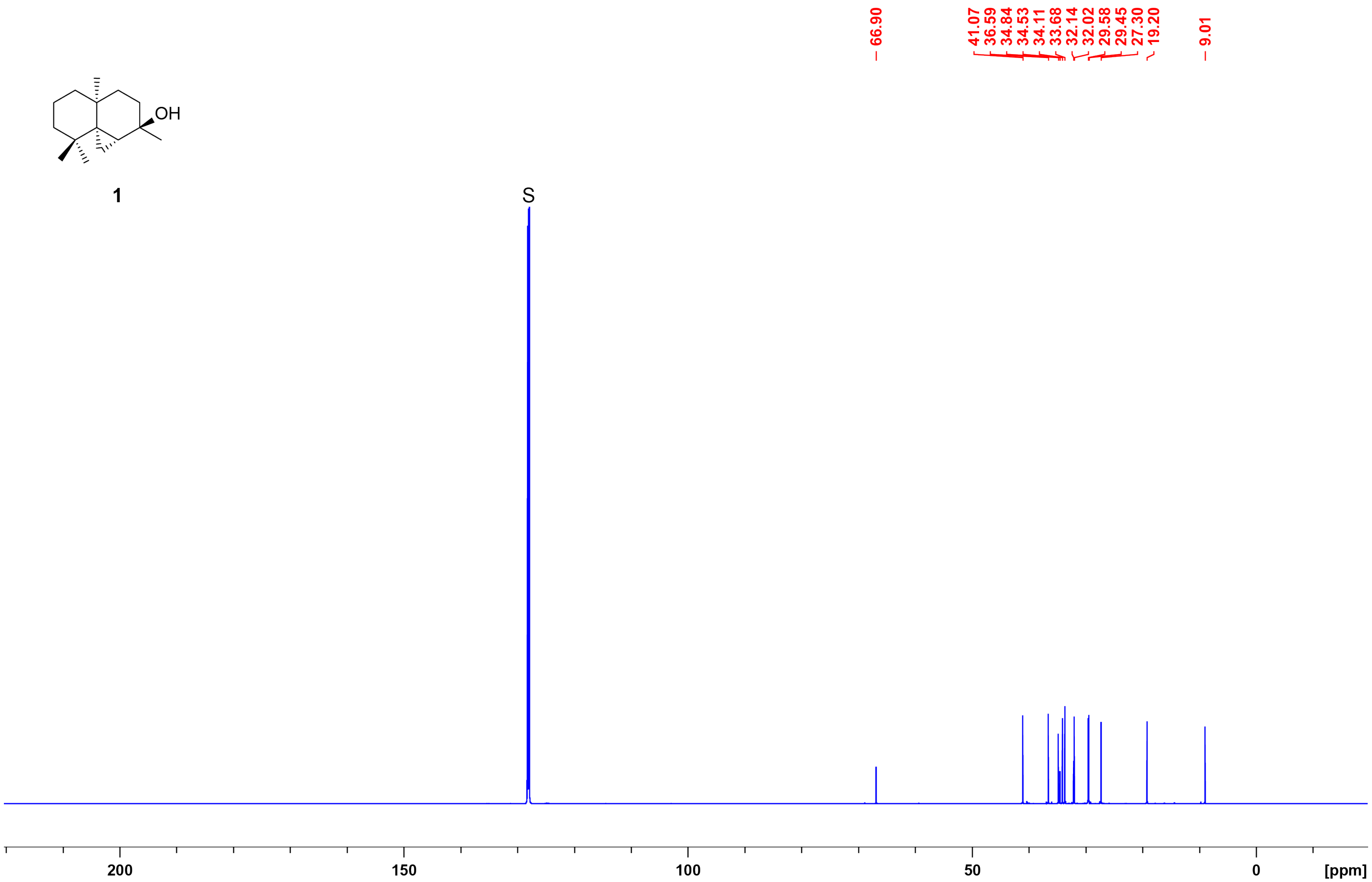

Figure S4. ^{13}C NMR spectrum of **1** (150 MHz, C_6D_6). The signal marked "S" indicates the residual solvent peak.

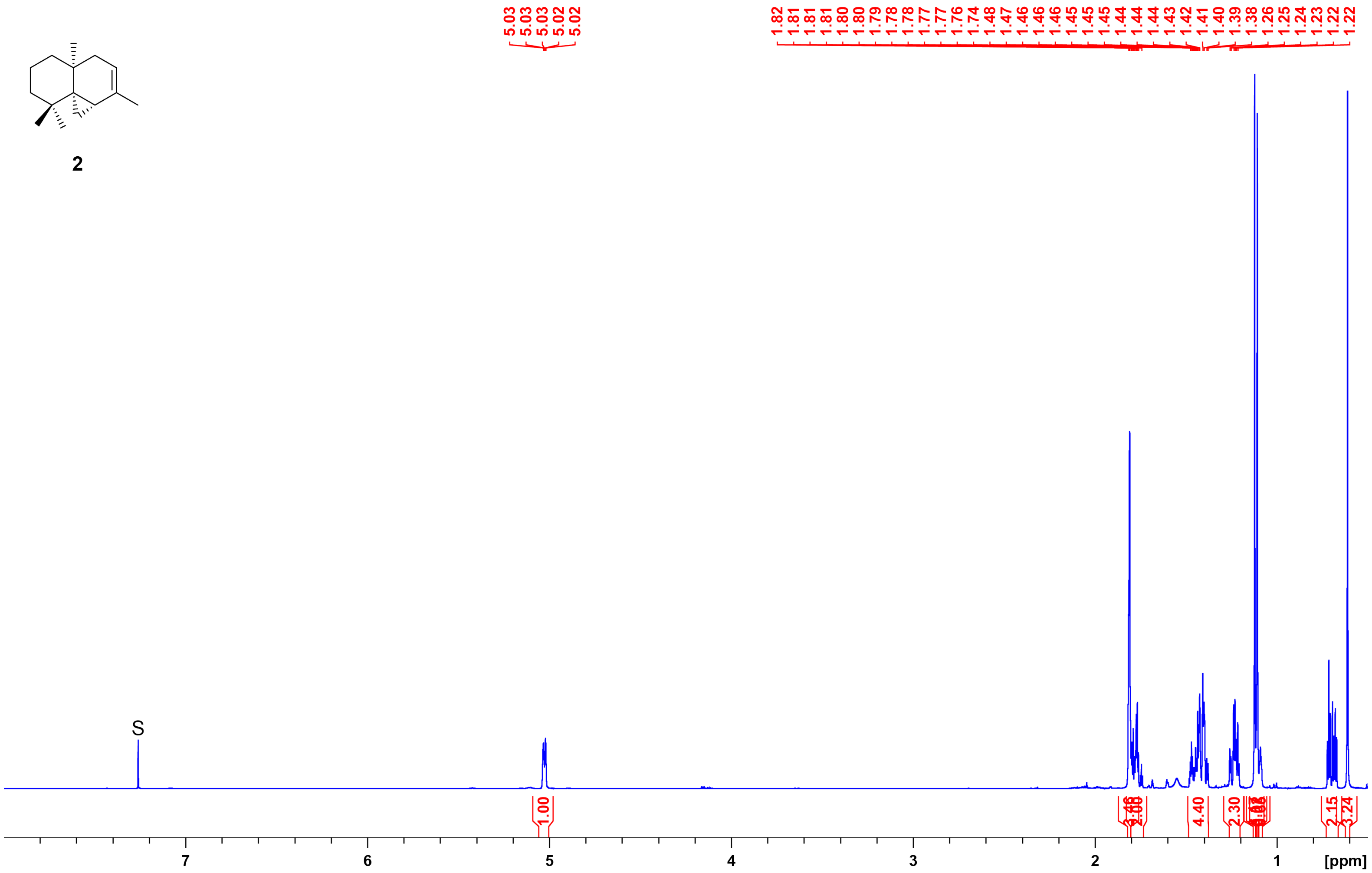

Figure S5. ^{1}H NMR spectrum of **2** (600 MHz, $CDCl_3$). The signal marked "S" indicates the residual solvent peak.

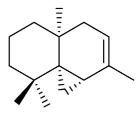

2

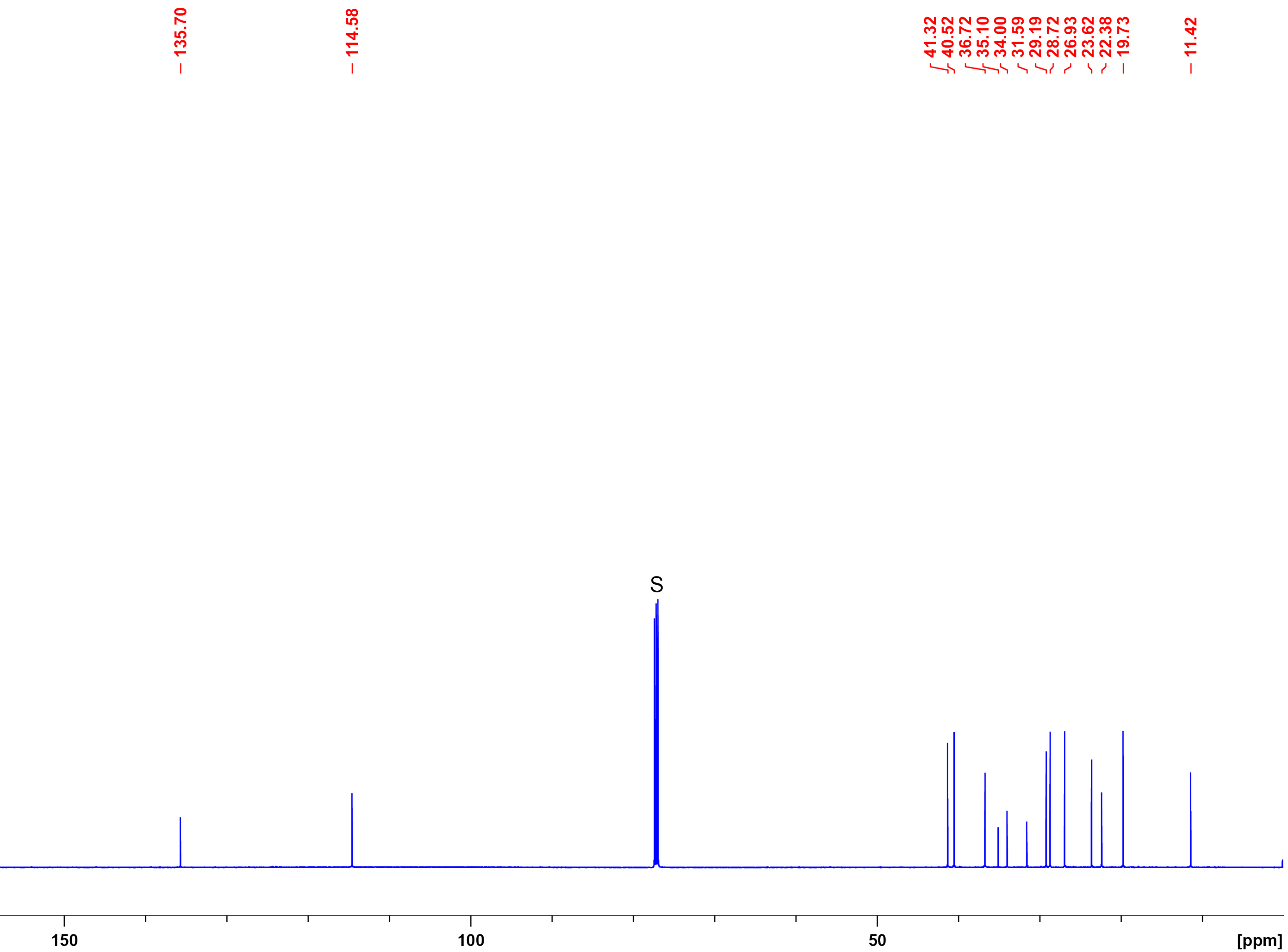

Figure S6. ^{13}C NMR spectrum of **2** (150 MHz, $CDCl_3$). The signal marked "S" indicates the residual solvent peak.

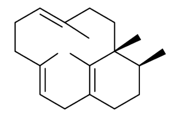

3

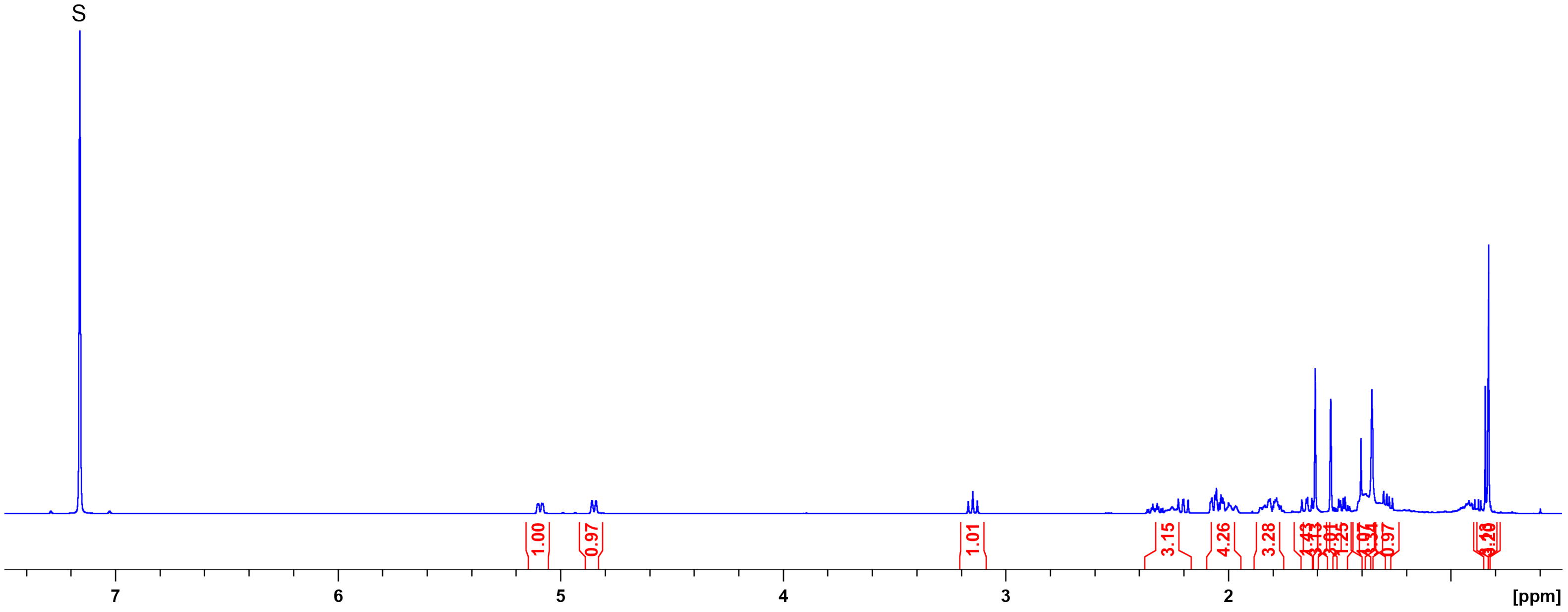

Figure S7. ^{1}H NMR spectrum of **3** (600 MHz, C_6D_6). The signal marked “S” indicates the residual solvent peak.

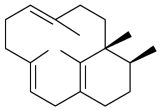

3

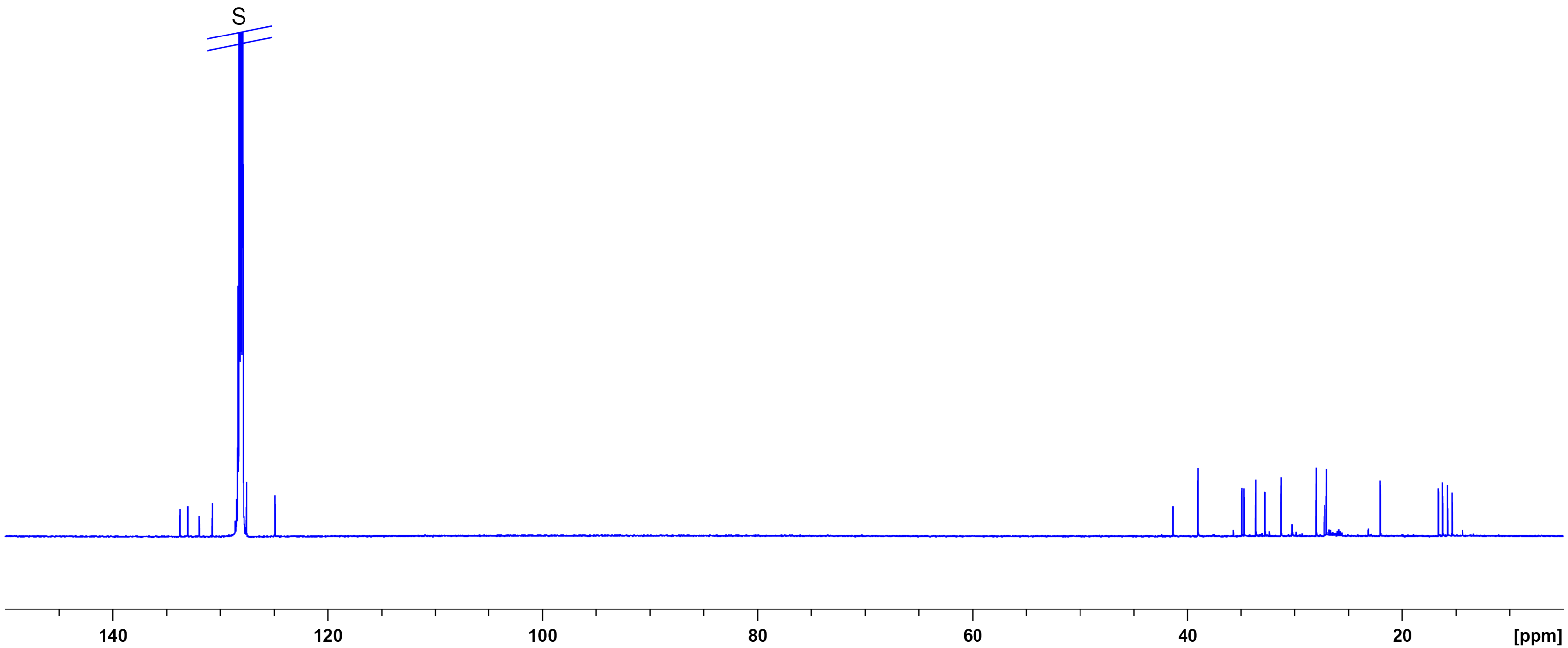

Figure S8. ^{13}C NMR spectrum of **3** (150 MHz, C_6D_6). The signal marked "S" indicates the residual solvent peak.

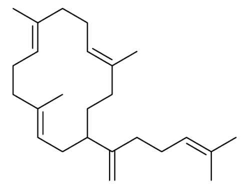

5

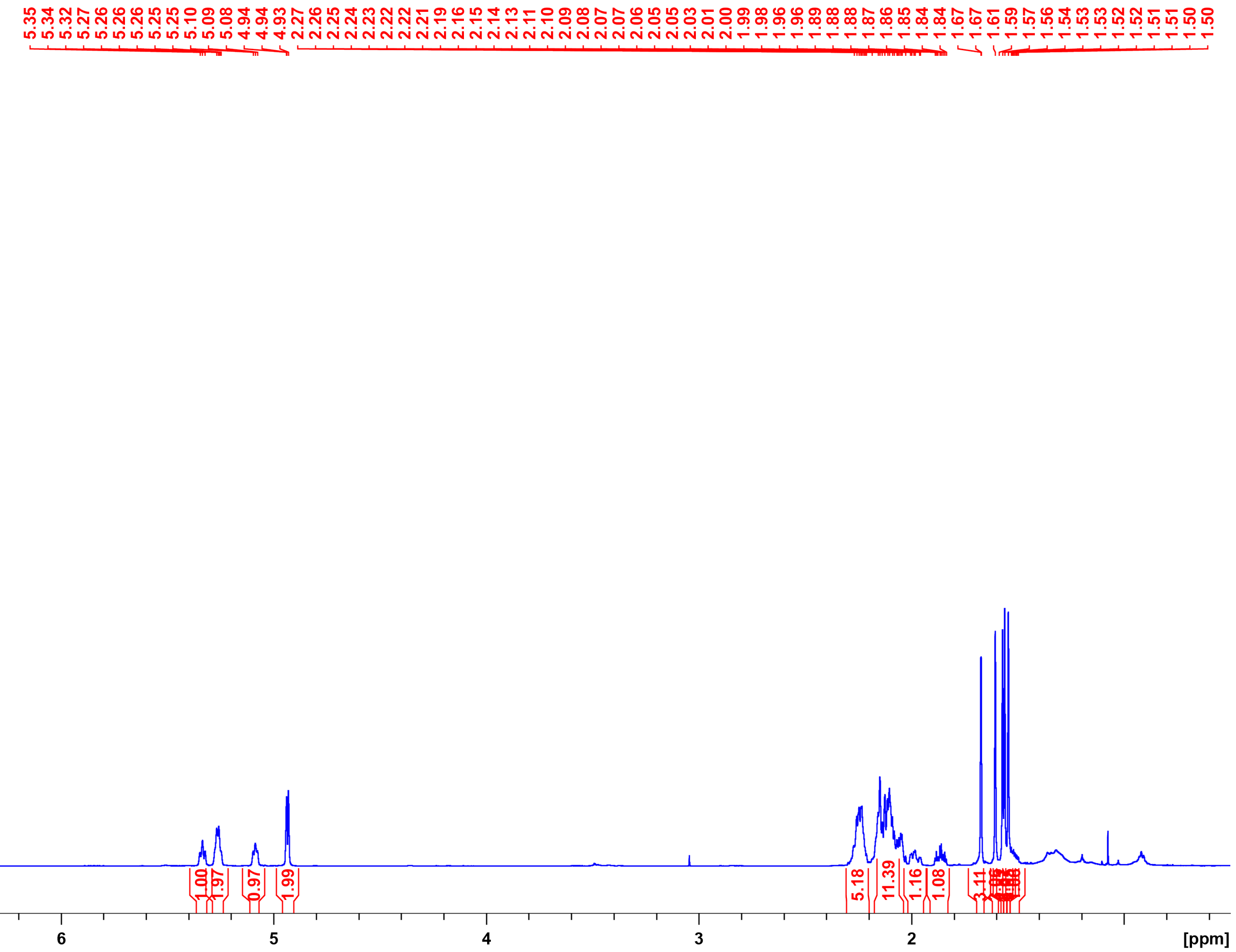

Figure S9. ^{1}H NMR spectrum of **5** (600 MHz, C_6D_6). The signal marked “S” indicates the residual solvent peak.

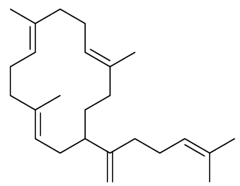

5

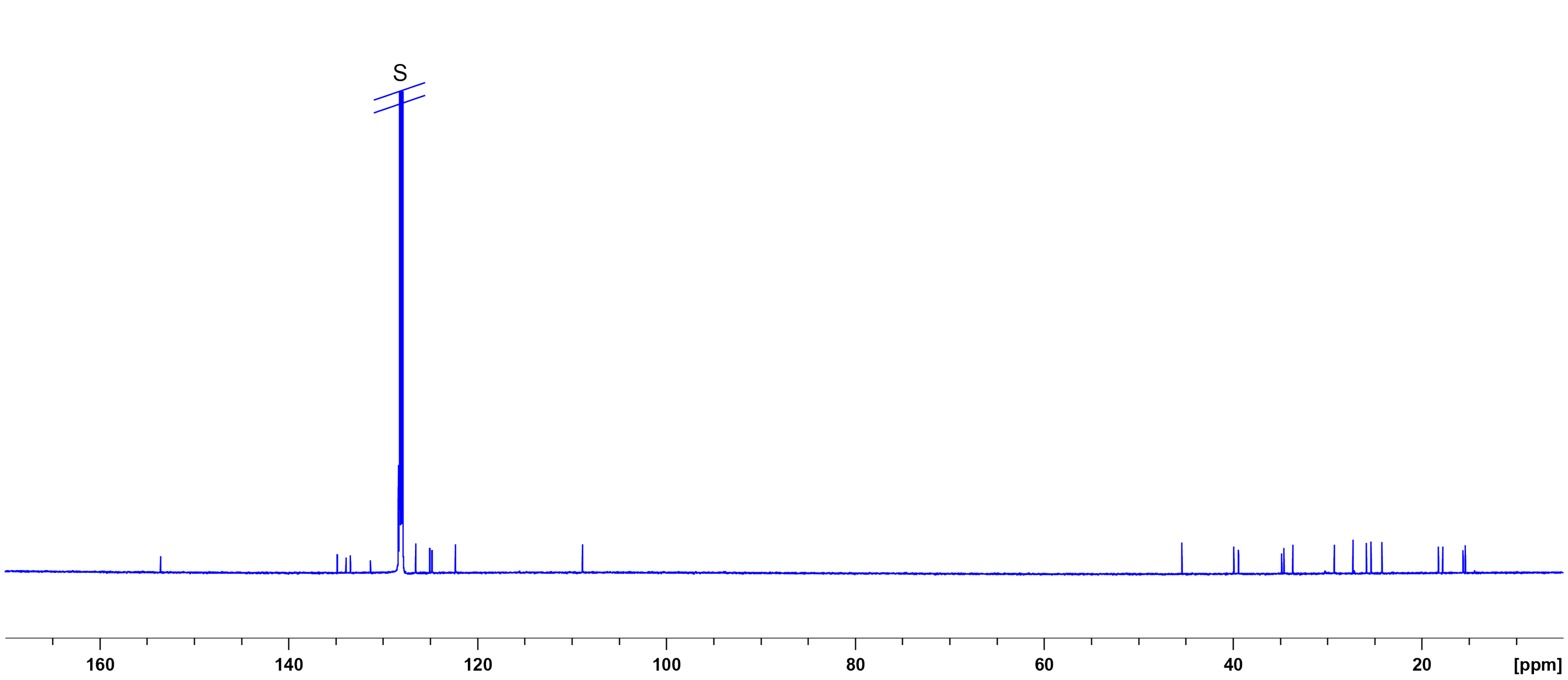

Figure S10. ^{13}C NMR spectrum of **5** (150 MHz, C_6D_6). The signal marked "S" indicates the residual solvent peak.

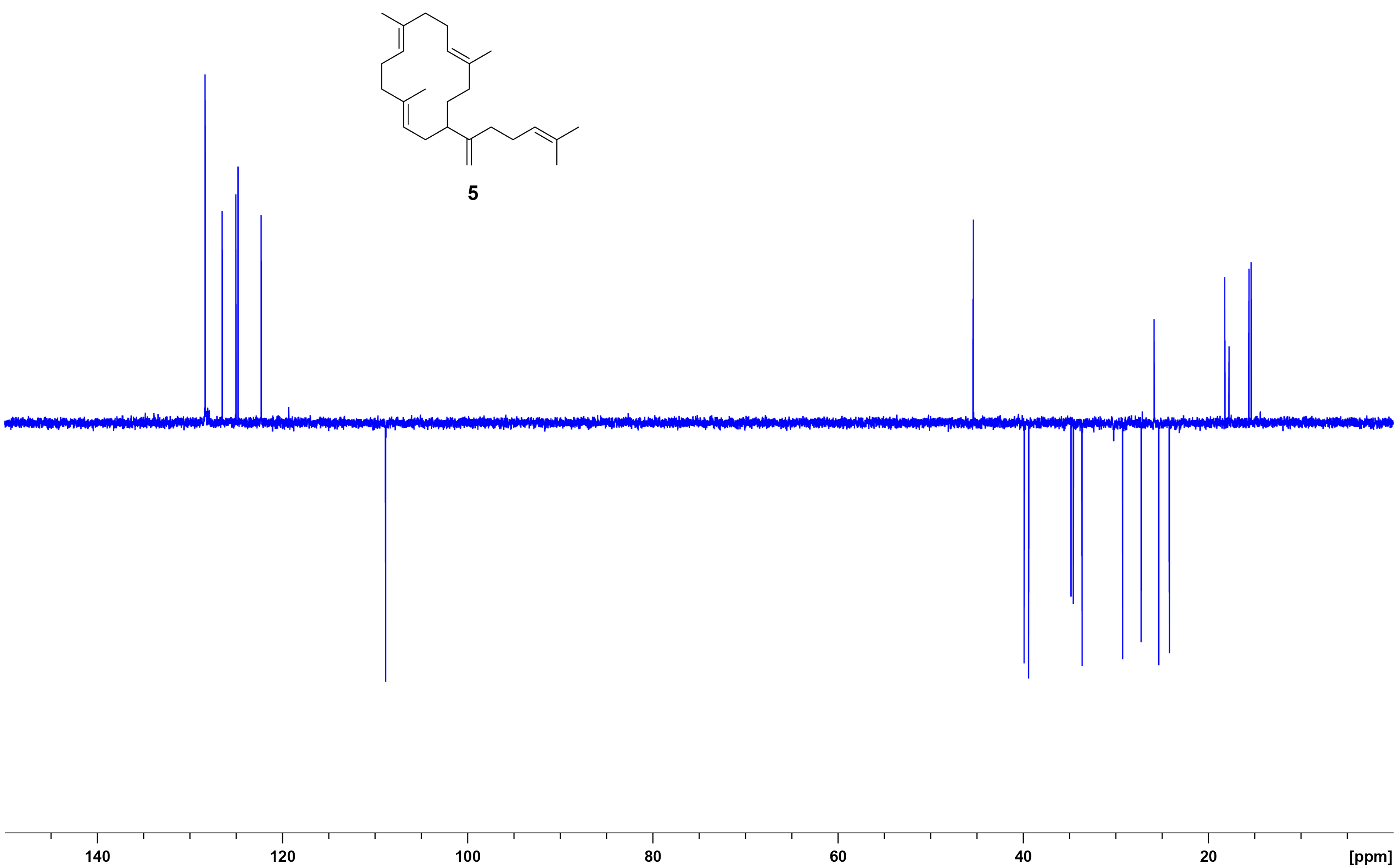

Figure S11. ^{13}C DEPT135 spectrum of **5** (150 MHz, C_6D_6).

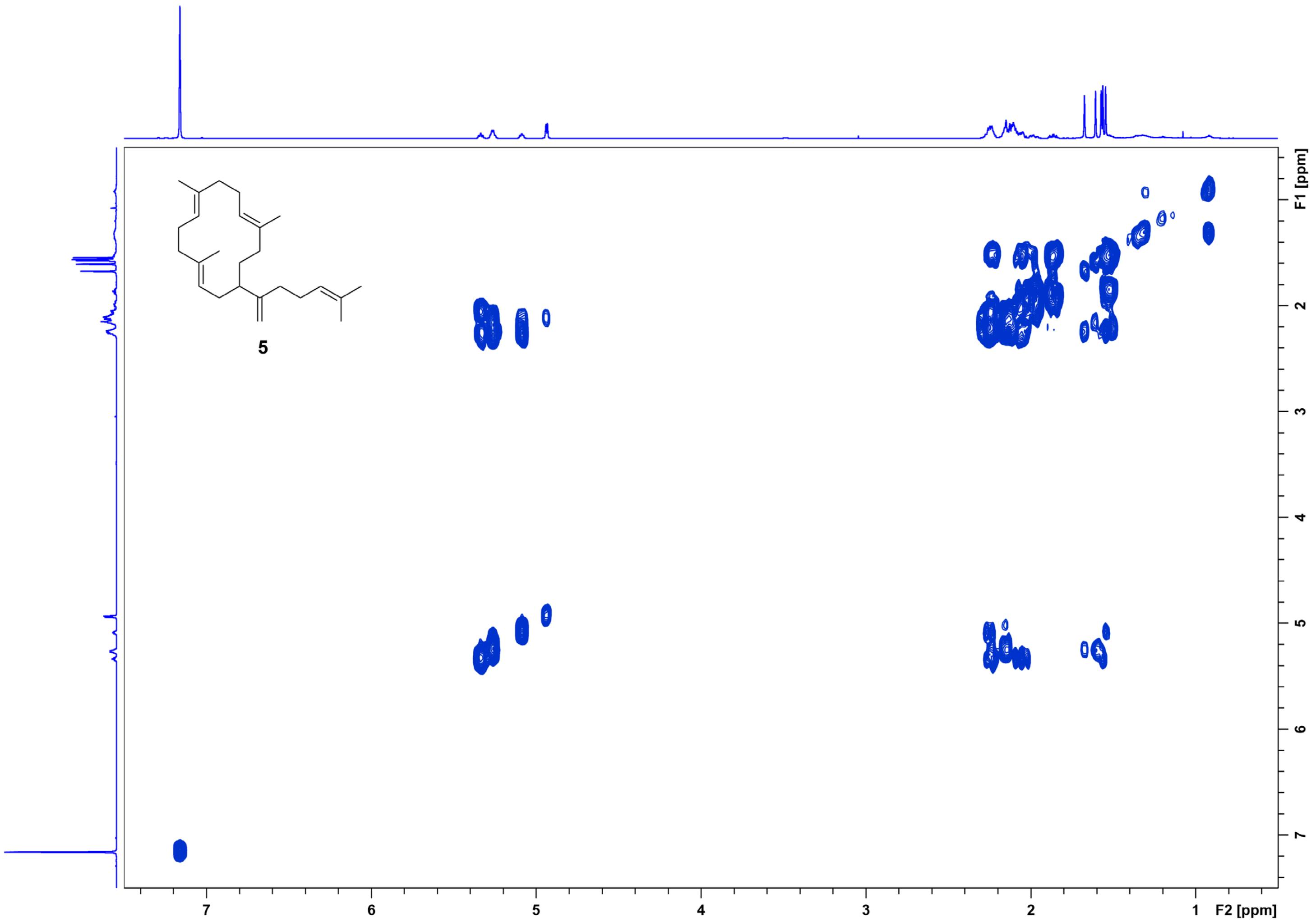

Figure S12. ^{1}H-^{1}H COSY spectrum of **5** (C_6D_6).

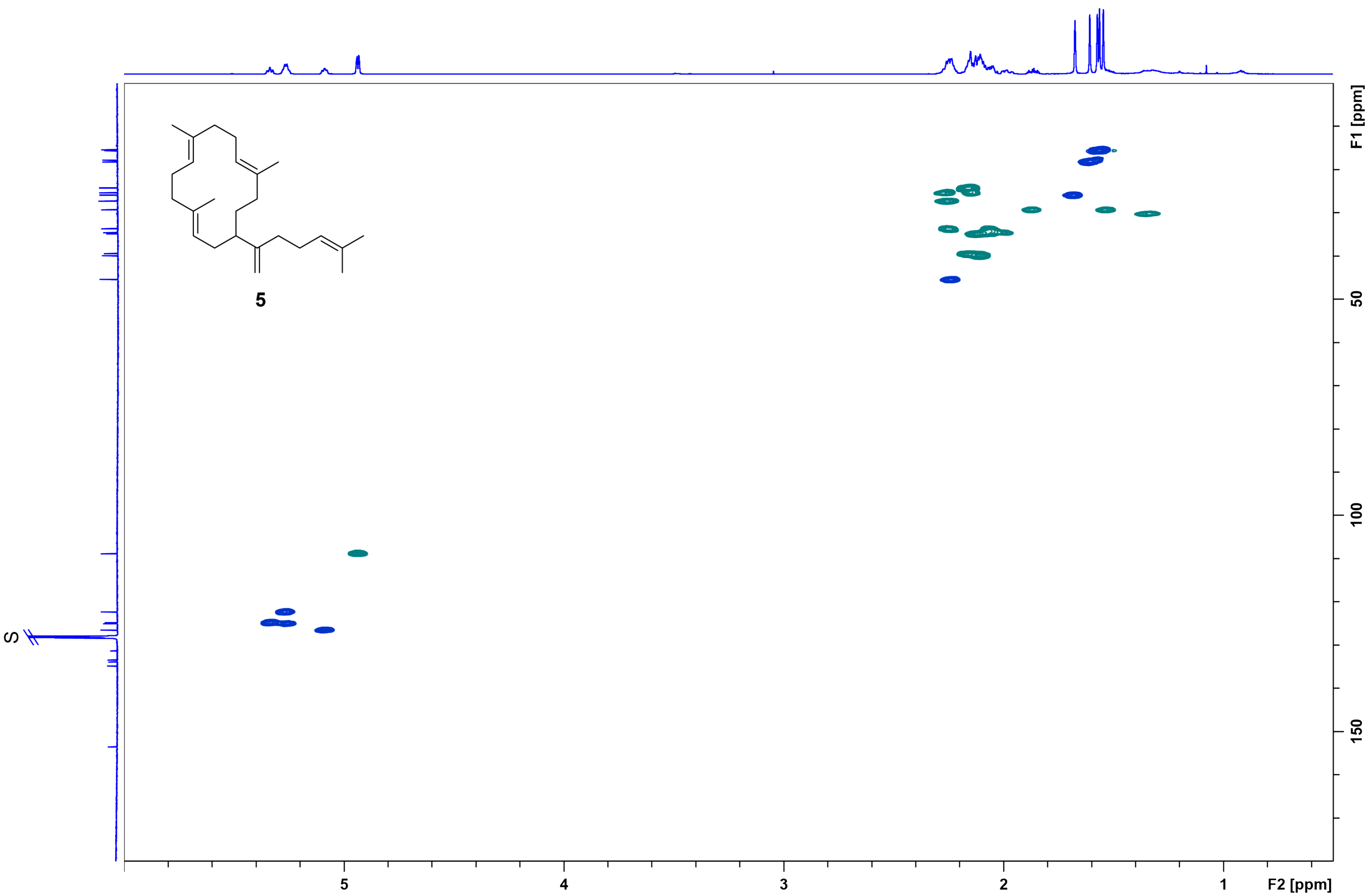

Figure S13. HSQC spectrum of **5** (C_6D_6).

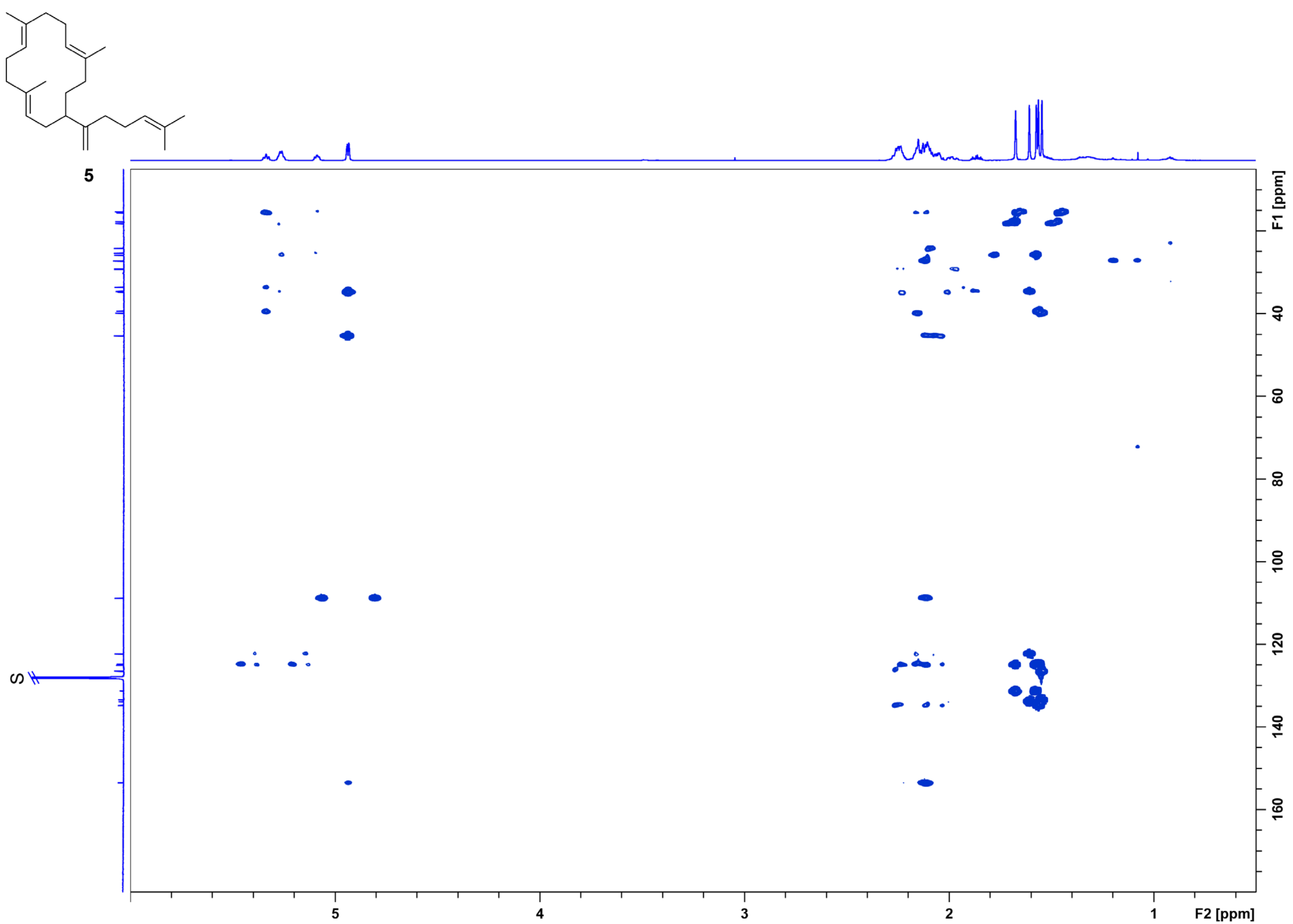

Figure S14. HMBC spectrum of **5** (C_6D_6).

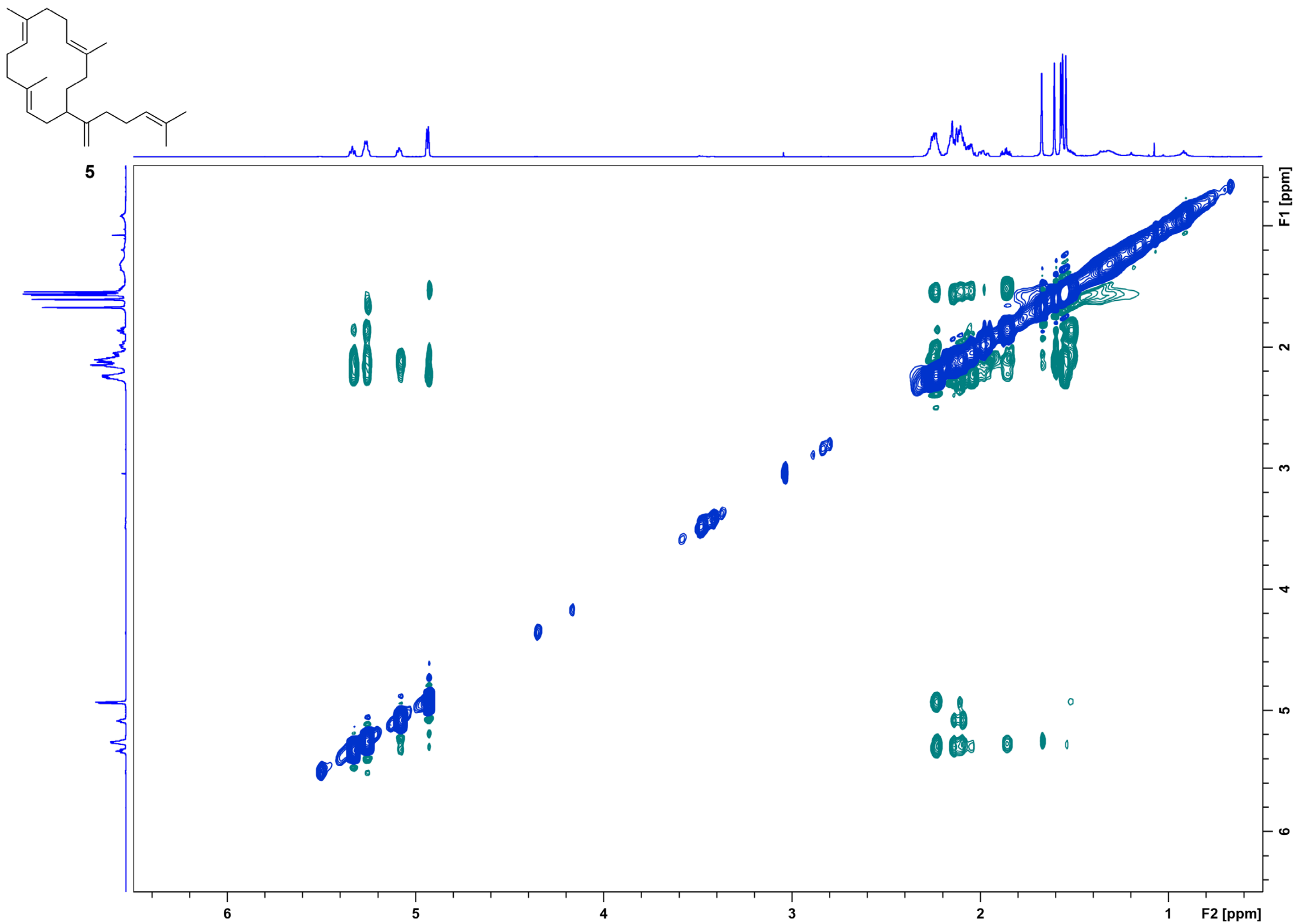

5

Figure S15. NOESY spectrum of **5** (C_6D_6).

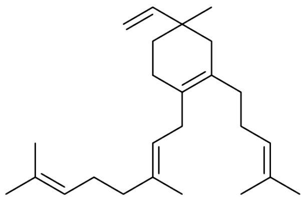

6

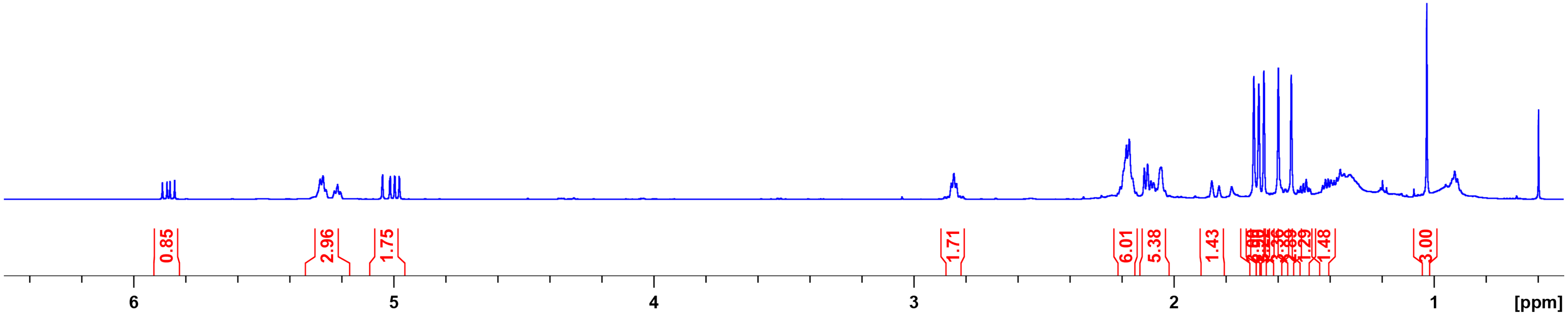

Figure S16. ^{1}H NMR spectrum of **6** (600 MHz, C^6D_6).

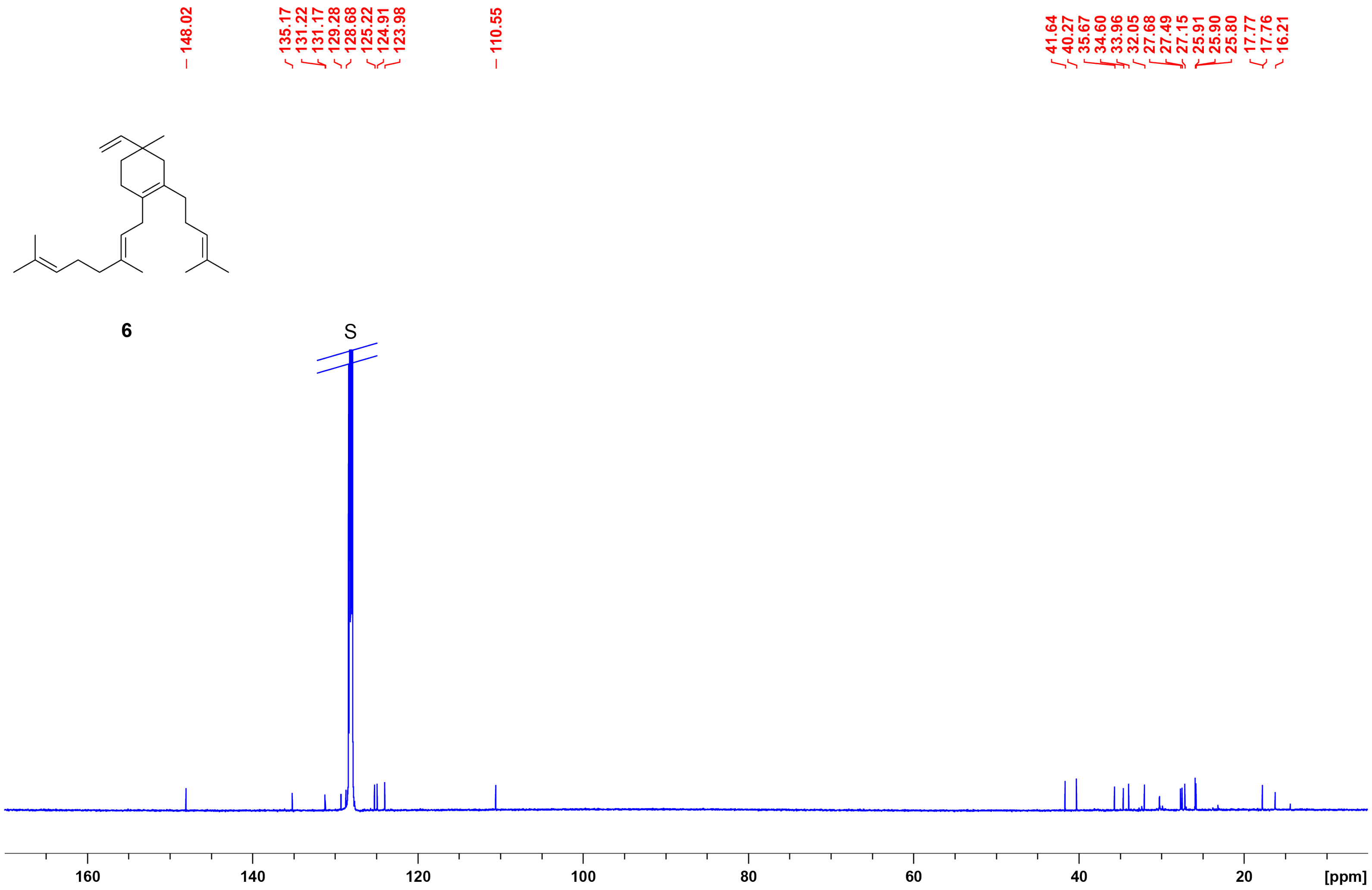

Figure S17. ^{13}C NMR spectrum of **6** (150 MHz, C_6D_6). The signal marked "S" indicates the residual solvent peak.

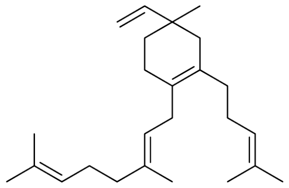

6

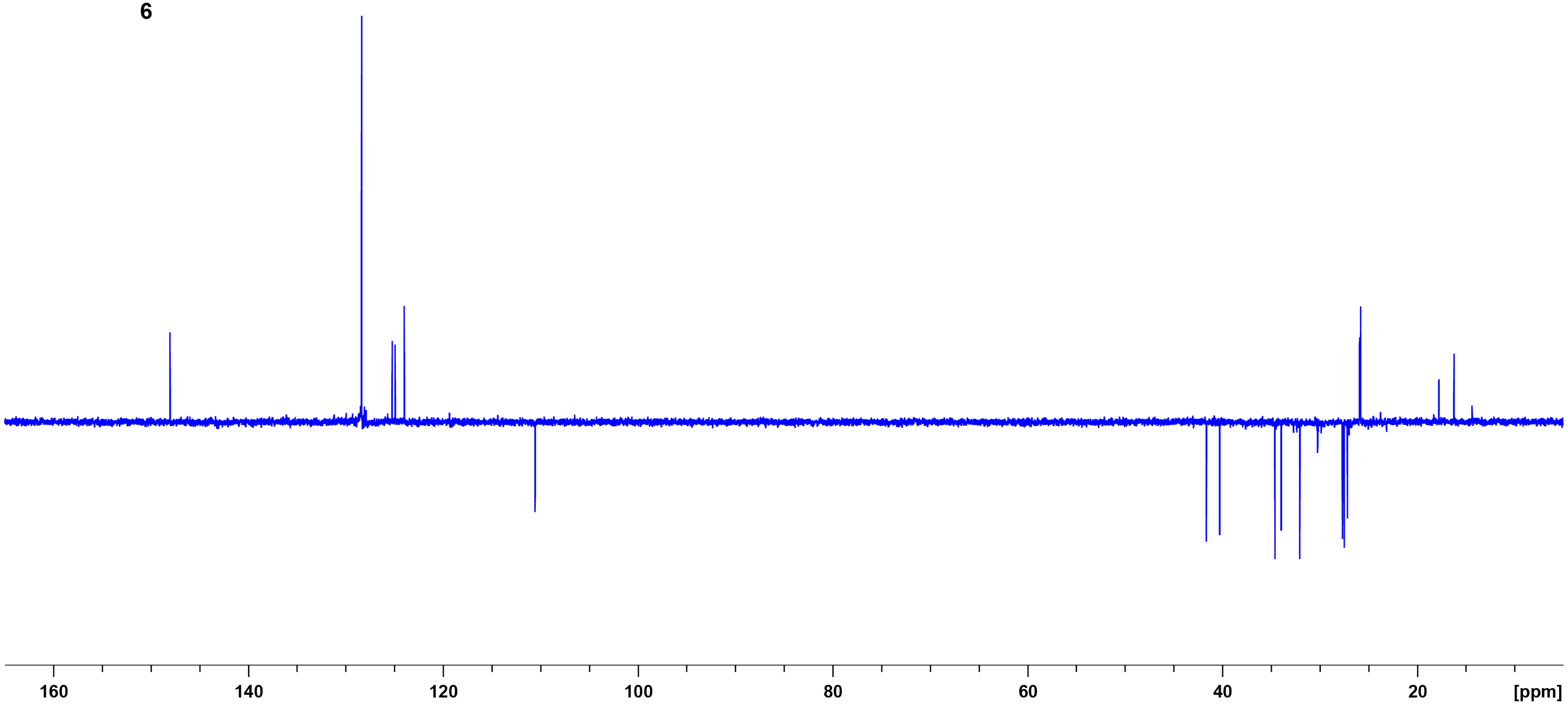

Figure S18. ^{13}C DEPT135 spectrum of **6** (150 MHz,C_6D_6).

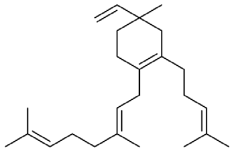

6

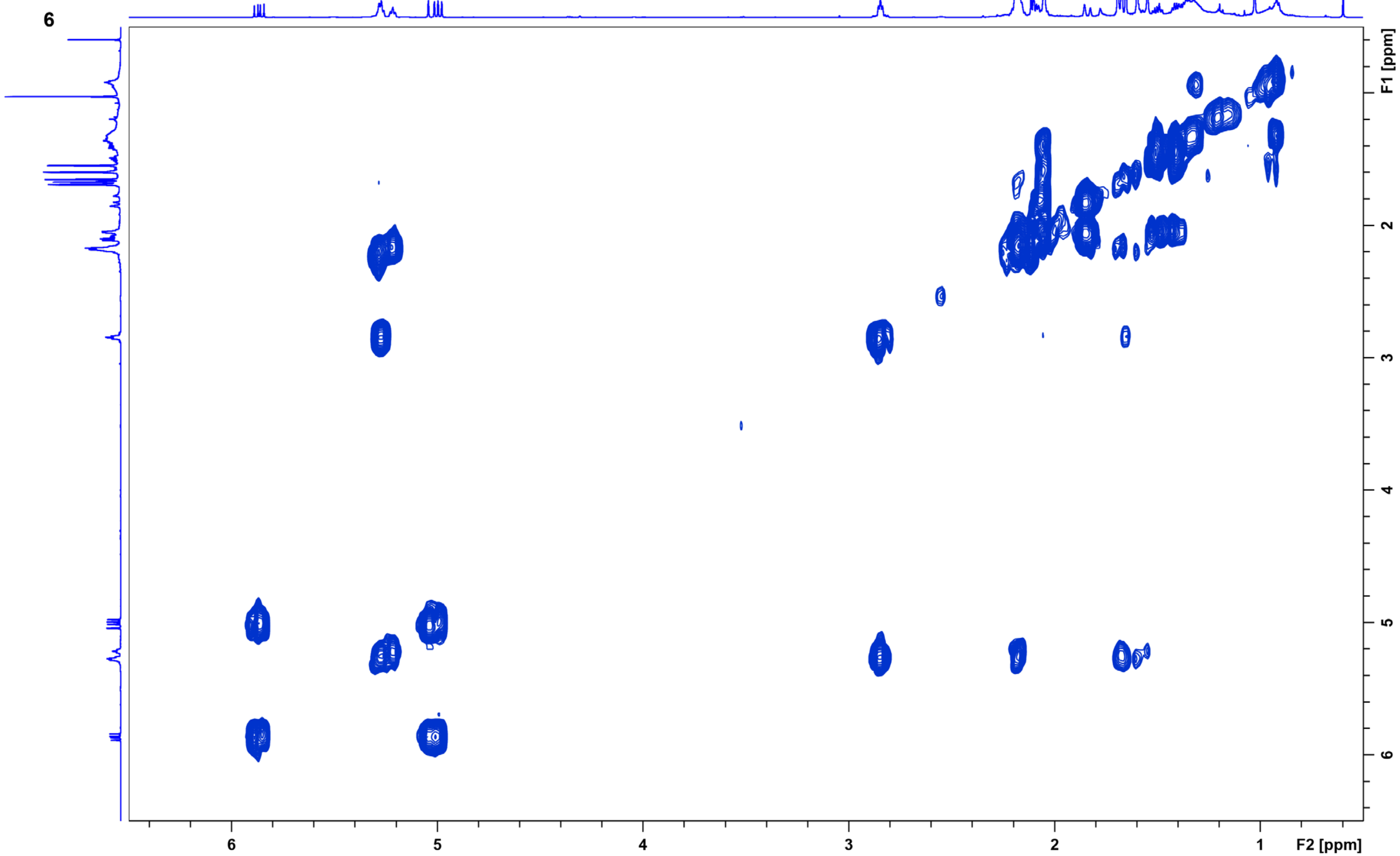

Figure S19. ^{1}H-^{1}H COSY spectrum of **6** (C_6D_6).

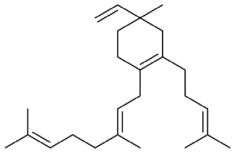

6

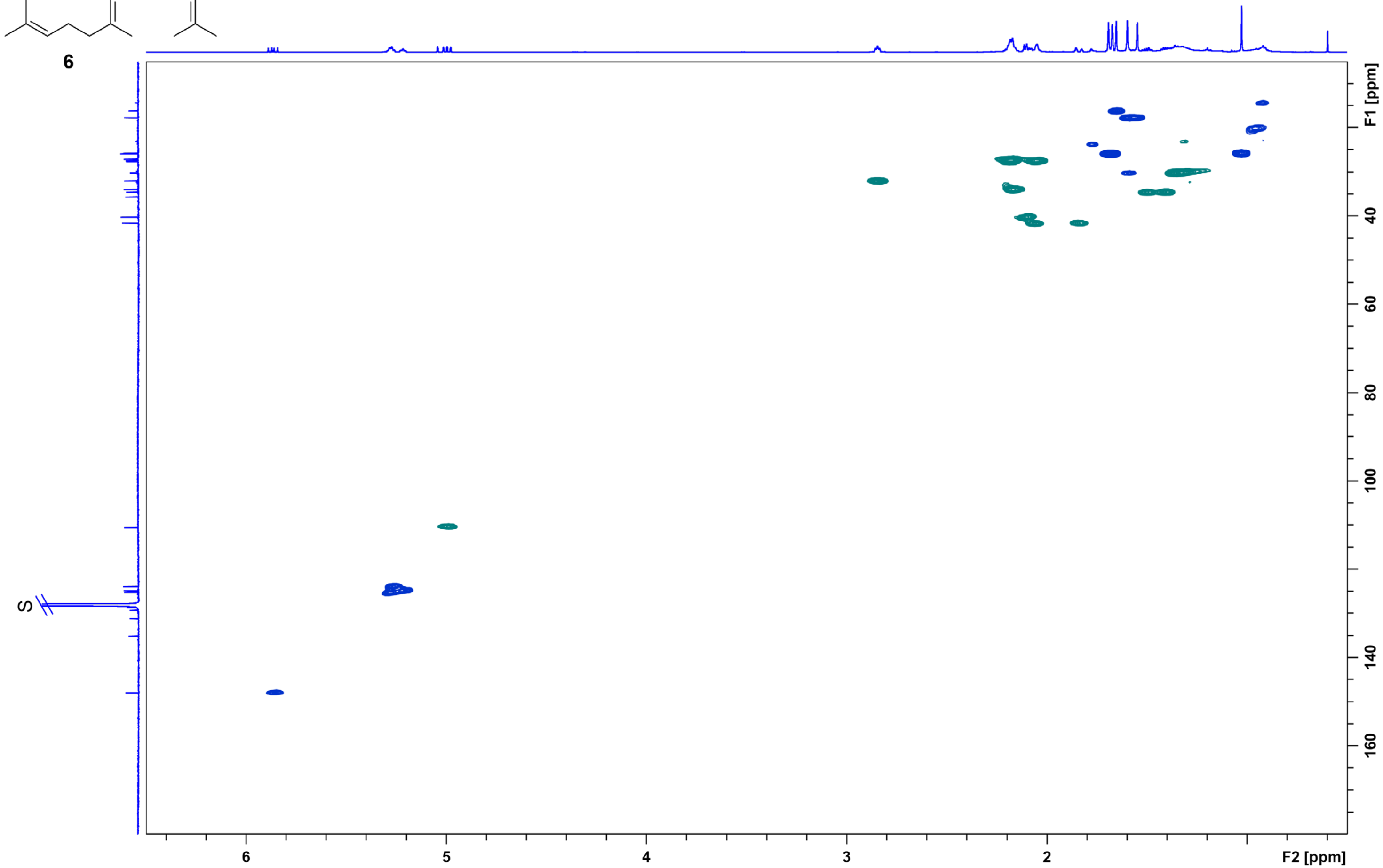

Figure S20. HSQC spectrum of **6** (C_6D_6).

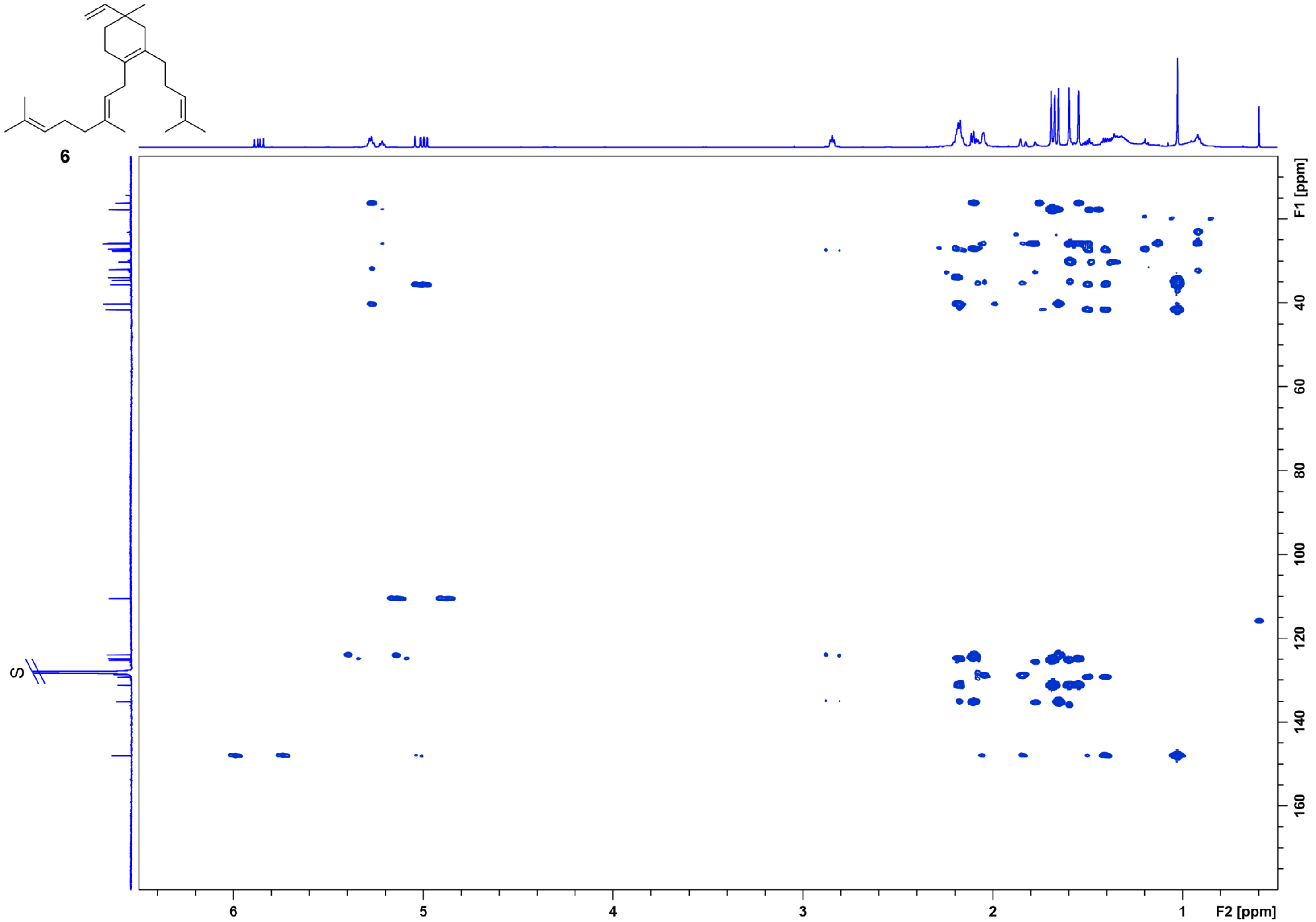

Figure S21. HMBC spectrum of **6** (C_6D_6).

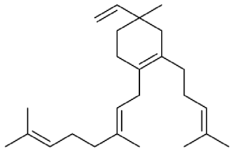

6

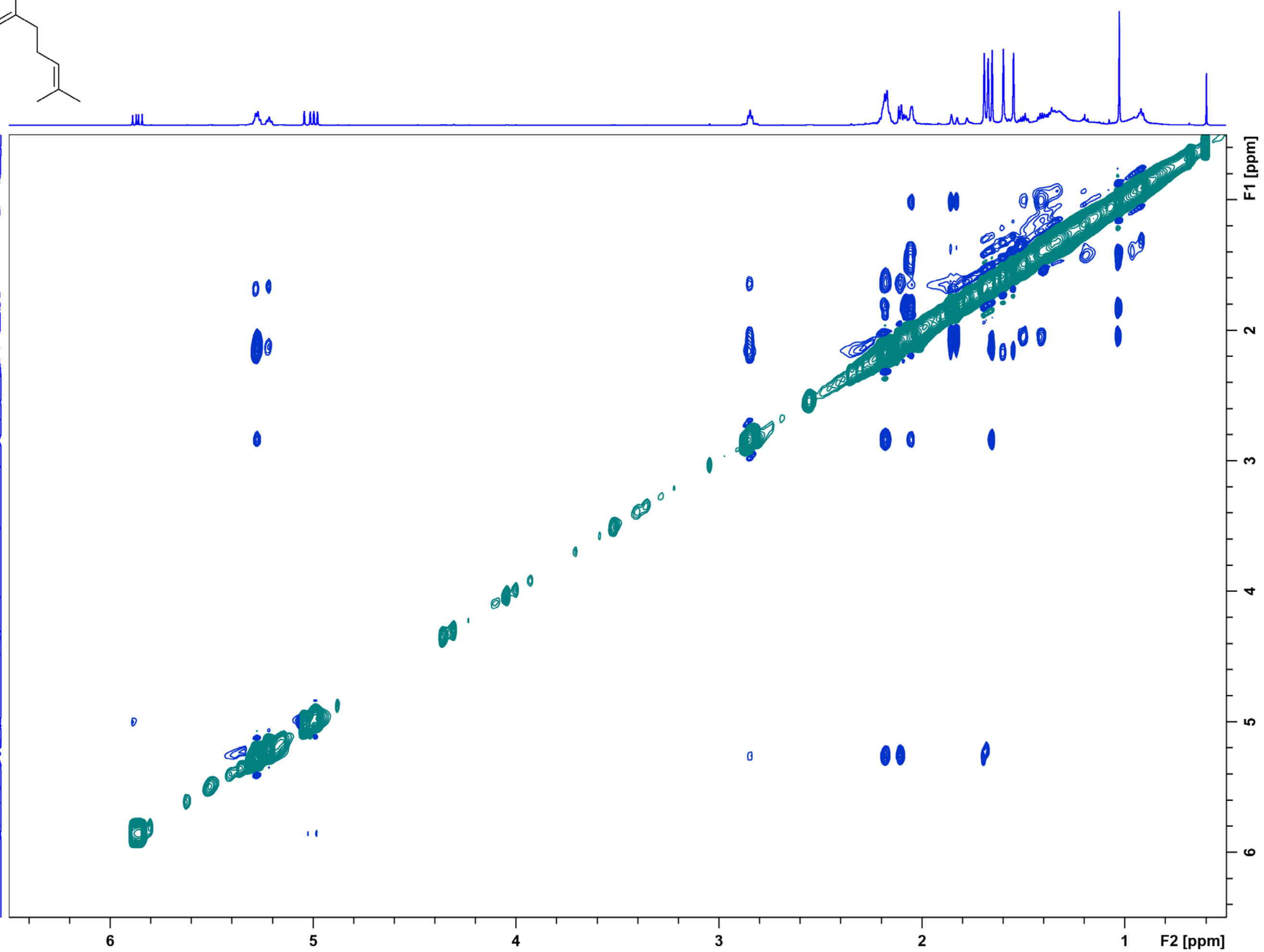

Figure S22. NOESY spectrum of **6** (C_6D_6).

Table S3. Calculated energies and Boltzmann populations of **6** conformers in benzene.

Conformers	Energy (Hartree)	ΔE (Hartree)	ΔE (kcal/mol)	Filtered_ Boltzmann Population (%)a
M0003	-976.294	0	0	38.7611473
M0008	-976.292	0.001122	0.704065	11.8102822
M0001	-976.292	0.001441	0.90424	8.42394698
M0007	-976.292	0.001451	0.910516	8.33518952
M0004	-976.292	0.001618	1.01531	6.98382013
M0022	-976.292	0.001866	1.170932	5.37043459
M0019	-976.291	0.002113	1.325927	4.13414621
M0018	-976.291	0.00219	1.374245	3.8103486
M0020	-976.291	0.002256	1.41566	3.55306987
M0002	-976.291	0.00234	1.468371	3.25059247
M0005	-976.291	0.002403	1.507904	3.04075624
M0012	-976.291	0.002578	1.617718	2.52626588
